# Decoupling of Light and Dark Reactions in a 2D Niobium Tungstate for Light-Induced Charge Storage and On-Demand Hydrogen Evolution

**DOI:** 10.1021/jacs.4c04140

**Published:** 2024-09-04

**Authors:** Yang Wang, Yu-Te Chan, Takayoshi Oshima, Viola Duppel, Sebastian Bette, Kathrin Küster, Andreas Gouder, Christoph Scheurer, Bettina V. Lotsch

**Affiliations:** † Max Planck Institute for Solid State Research, Stuttgart 70569, Germany; ‡ Theory Department, Fritz-Haber-Institut der Max-Planck-Gesellschaft, Berlin 14195, Germany; § IEK-9, Forschungszentrum Jülich, Jülich D-52425, Germany; ∥ Department of Chemistry, Ludwig-Maximilians-Universität (LMU), Butenandtstr. 5-13, Munich 81377, Germany; ⊥ e-conversion, Lichtenbergstr. 4a, Garching 85748, Germany

## Abstract

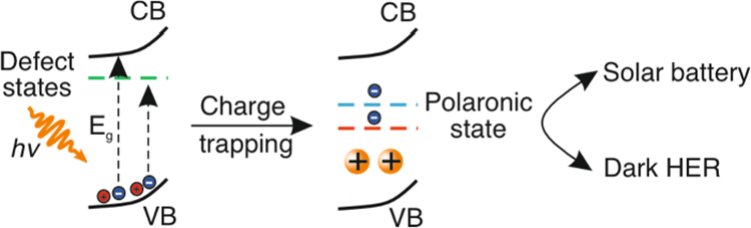

The direct coupling of light harvesting and charge storage in a single material opens new avenues to light storing devices. Here we demonstrate the decoupling of light and dark reactions in the two-dimensional layered niobium tungstate (TBA)^+^(NbWO_6_)^−^ for on-demand hydrogen evolution and solar battery energy storage. Light illumination drives Li^+^/H^+^ photointercalation into the (TBA)^+^(NbWO_6_)^−^ photoanode, leading to small polaron formation assisted by structural distortions on the WO_*x*_ sublattice, along with a light-induced decrease in material resistance over 2 orders of magnitude compared to the dark. The photogenerated electrons can be extracted on demand to produce solar hydrogen upon the addition of a Pt catalyst. Alternatively, they can be stored for over 20 h under oxygen-free conditions after 365 nm UV illumination for only 10 min, thus featuring a solar battery anode with promising capacity and long-term stability. The optoionic effects described herein offer new insights to overcome the intermittency of solar irradiation, while inspiring applications at the interface of solar energy conversion and energy storage, including solar batteries, “dark” photocatalysis, solar battolyzers, and photomemory devices.

## Introduction

The direct conversion of solar energy into electrical energy and chemical fuels are two promising approaches to mitigate our reliance on fossil fuels and to expedite the energy transition. However, the intermittency of solar irradiation is a major bottleneck whose compensation can lead to a temporary shortage of the renewable energy supply and a critical load on the grid. Among the solutions, emerging renewable energy concepts such as direct solar batteries and on-demand “dark” (memory) photocatalysis show tremendous promise in this regard.^1−3^ Bifunctional solar batteries in which the photoelectrode both harvests light and stores charge typically utilize the process of photointercalation of ions into the host structure under light illumination. Light is thus directly stored in the form of electrochemical energy, followed by charge release by electric discharging.^4−8^ Similarly, on-demand dark photocatalysis decouples solar energy storage and release to produce chemical fuels like hydrogen upon the addition of a catalyst in the dark.^2,9,10^ This process mimics natural photosynthesis in that it separates the light-dependent from the light-independent reactions, thus providing a blueprint for overcoming the intermittency of solar irradiation.

To minimize energy loss and cost and to maximize integration and compactness, the ideal light storing system would combine solar energy storage and release within a single material. Different materials have been reported either as photoelectrodes for solar batteries including V_2_O_5_,^11,12^ perovskites,^13^ MoO_3_,^14^ TiO_2_,^15^ 2D potassium poly(heptazine imide) (K-PHI),^1,3^ and covalent organic frameworks,^16−18^ or as dark hydrogen evolution (HER) materials, including K-PHI,^2,10^ MOF-253,^19^ MIL-125,^20^ and photosensitizer-polyoxometalate couples.^9^ Among them, dual functionality as solar battery photoanode and dark hydrogen evolution material has only been demonstrated for the 2D ionic carbon nitride so far.^1,2^ Upon light illumination, photogenerated electrons in K-PHI are trapped in the form of stable π-radicals, which are screened and charge-compensated by photointercalation of K^+^ ions—an optoionic process coupling light absorption with ion uptake. However, the intrinsically low electronic conductivity of this 2D carbon nitride hampers charge transfer within K-PHI and to the substrate, thus leading to increased recombination and limiting charge carrier collection. The resulting limitations in terms of capacity utilization and (dis)charging kinetics call for the search for alternative bifunctional semiconductor materials for light storage applications with improved charge trapping and charge carrier transport (both electronic and ionic) characteristics. At the same time, in order to rationally develop tailor-made optoionic materials, a better understanding and control of the mechanism of charge trapping is critical.

Recently, Cronin and co-workers established polyoxometallates such as [P_2_W_18_O_62_]^6–^ as dual functional redox systems that can either act as a redox-flow battery electrolyte or as a mediator in an electrolytic cell for hydrogen generation.^21^ Likewise, Mulder et al. demonstrated a Ni(OH)_2_/Fe(OH)_2_-based battery with built-in hydrogen and oxygen evolving capability.^22^ Such battery—electrolyzers (“battolysers”) form a new generation of integrated charge storing and fuel generating devices, which can act as flexible buffer systems that are charged and provide electricity when surplus renewable electricity is available (battery functionality), and that convert electricity into hydrogen as an alternative long-term chemical storage (fuel functionality), thus providing intermittency-adapted energy utilization. In this layout, electricity is fed into the battolyzer from remote renewable resources such as PV or wind power. In contrast, the direct storage of solar energy in a dual functional “solar battolyzer” would represent yet another level of integration and another stepping stone toward a more flexible and compact energy infrastructure.

**Figure 1:**
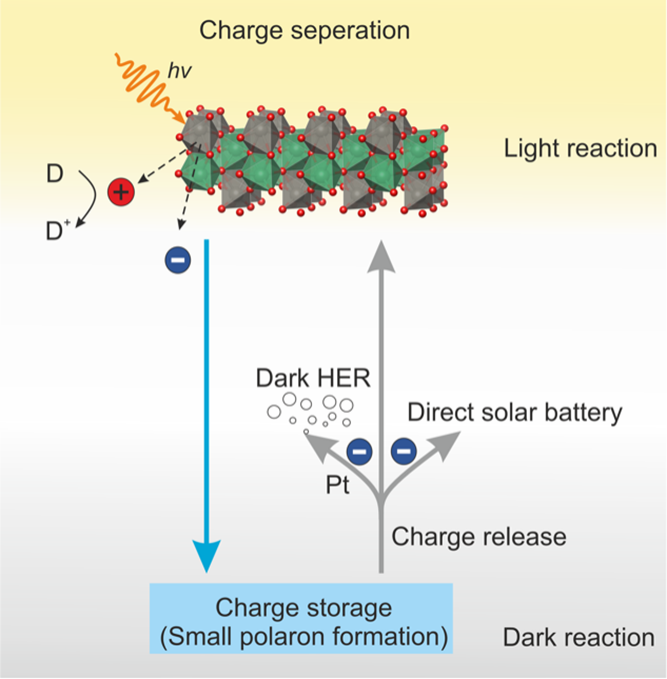
Schematic illustration of the decoupling of the light reaction and dark reaction of solar energy conversion. In the light reaction, above-band gap irradiation of NbWO_6_ creates electron–hole pairs, where the hole is quenched by an electron donor (D) and the electron is trapped locally to form a small polaron–cation complex. The photogenerated electron is thus stabilized and stored and can be extracted with a time delay for on-demand applications in the dark (dark reaction). One example is a direct solar battery, which can extract charge by applying current to electrically discharge the material. The other example is dark HER. After adding a Pt nanoparticle catalyst in the dark, the photogenerated electron is transferred to the Pt catalyst to generate hydrogen on-demand. Finally, the whole system is recycled and the process can start over. The red sphere/circle denotes the hole, and blue the electron; *h*, Planck’s constant; ν, frequency.

Following this thought, here we introduce the n-type 2D semiconductor (TBA)^+^(NbWO_6_)^–^ (abbreviated as NbWO_6_ in the following)^23^ as a candidate “solar battolyzer” material. Films fabricated from ultrathin, redox-active NbWO_6_ nanosheets are able to simultaneously harvest and store solar energy as electricity for short-term storage, and convert it into hydrogen for long-term storage; they can thus be used as both direct solar battery photoanode and for on-demand hydrogen generation in conjunction with a Pt catalyst (Figure 1). The high stability of photogenerated, trapped electrons suggests the efficient formation of small polarons in NbWO_6_, which is confirmed by our DFT results. Based on this mechanism, NbWO_6_ effectively decouples the light and dark reactions of solar energy conversion and represents a new prototype material that combines both direct solar battery and fuel converting function in a single material, thus paving the way to future solar battolyzers.

## Results and Discussion

### 2D NbWO_6_ Characterization

**Figure 2:**
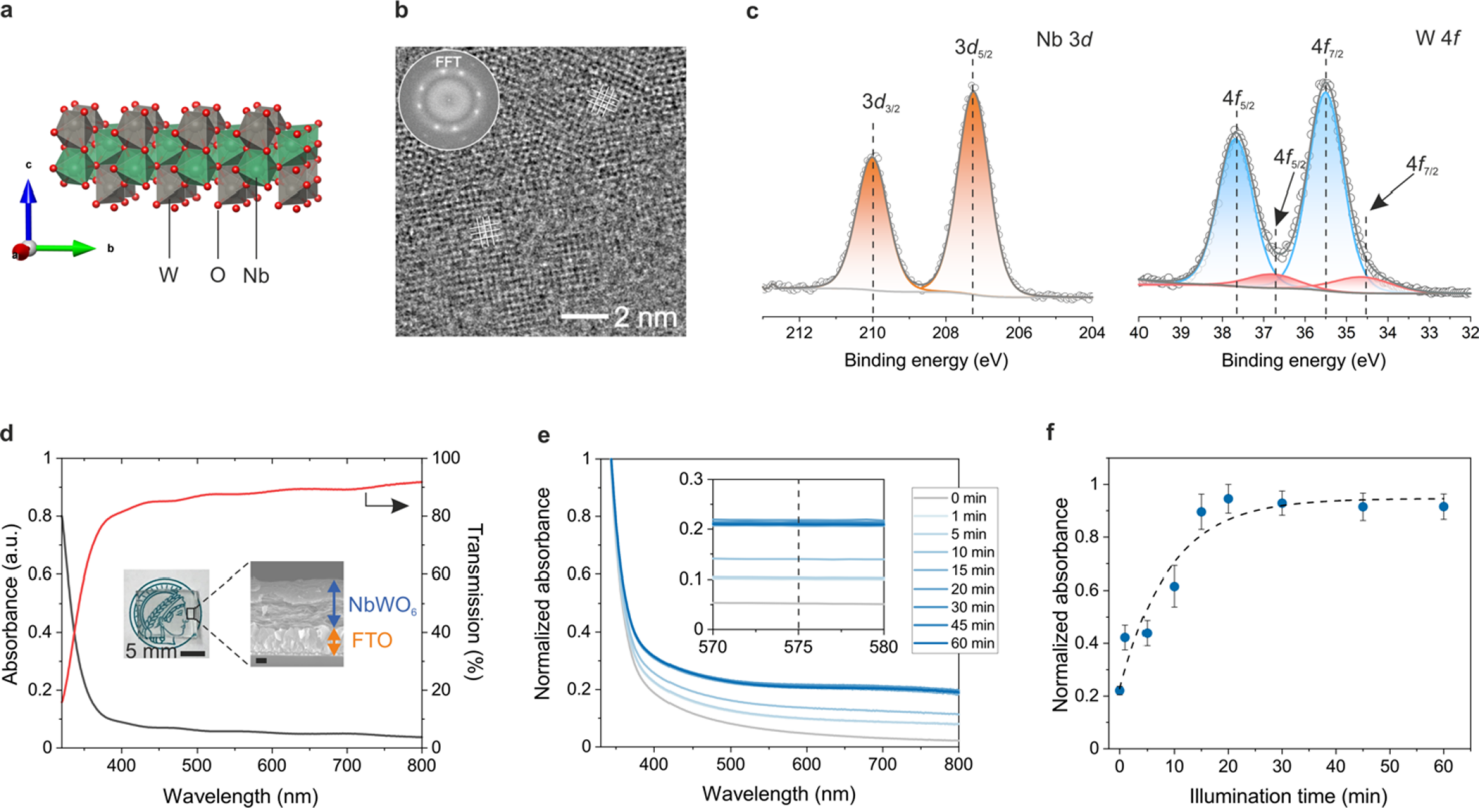
Structural and optical characterization of NbWO_6_nanosheets and film. (a) Crystal structure of a 2D NbWO_6_ nanosheet (TBA counter cations not shown). The gray and green polyhedra are WO_6_ and NbO_6_ octahedra, respectively; oxygen atoms in red. (b) HRTEM image of 2D NbWO_6_ restacked on a TEM grid. Insert at the top left is the FFT from the overlay of two sheets. (c) High-resolution XPS spectra of Nb 3d and W 4f in NbWO_6_ nanosheets deposited on a silicon substrate. (d) UV–vis transmission and absorption spectra of the NbWO_6_ thin film. Inset shows a digital photo (left) and cross-sectional SEM image (right, scale bar 200 nm) of the transparent NbWO_6_ film with a thickness around 870 nm on a FTO substrate. (e) Normalized operando UV–vis absorbance spectra of 1 mg mL^–1^ NbWO_6_ suspension in the presence of 10 vol % MeOH under 365 nm UV illumination for different times. The inset shows the magnified region between 570 and 580 nm. (f) Dependence of normalized absorbance intensity at 575 nm on illumination time. Values are means with standard deviation from three independent samples.

The 2D NbWO_6_ nanosheets were prepared by top-down liquid phase exfoliation of α-LiNbWO_6_ bulk powder. The pristine solid was first converted into HNbWO_6_·*x*H_2_O by protonation with HCl, followed by proton exchange with tetrabutylammonium hydroxide (TBAOH) solution, giving rise to (TBA)^+^(NbWO_6_)^−^ and water in a neutralization reaction (Figures S1–S7). After exfoliation, monolayer NbWO_6_ nanosheets were obtained that consist of layers of edge-sharing NbO_6_ and WO_6_ octahedra (Figure 2a).^24^ High-resolution transmission electron microscopy (HRTEM) and Fast Fourier Transform (FFT) of stacked sheets confirm the highly crystalline 2D structure of NbWO_6_ (Figures 2b and S6), while AFM reveals a thickness of the NbWO_6_ monolayer nanosheets between 1.4 and 1.9 nm (Figure S5), which is larger than the theoretical thickness of 7.19 Å obtained from the DFT relaxed NbWO_6_ model in the absence of solvent. This discrepancy in thickness can be ascribed to two main factors: first, the presence of charge compensating protons forming hydroxyl groups on both surfaces of the nanosheet, as well as the contribution of other species, here likely TBA^+^ for charge compensation. Second, hydrogen bonds may be formed between water and the nanosheet surface, which leads to an effective increase in the (solvated) nanosheet thickness.^25^ The NbWO_6_ surface composition and electronic structure were analyzed by X-ray photoelectron spectroscopy (XPS) as depicted in Figure 2c (Figure S8). While the Nb 3d XPS spectrum shows the presence of one Nb^5+^ species at 207.3 eV,^26^ the W 4f is compose of two different chemical species. The W 4f_7/2_ signal at 35.5 eV can be related to W^6+^,^27^ while the peak at 34.6 eV is related to W^5+^.^28^ The latter is likely due to native oxygen vacancies introduced during bulk synthesis or a reduction of some of the W^6+^ to W^5+^ during the XPS measurements (Figure S8). The ratio of W^6+^/W^5+^ is approximately 7:1. According to our DFT calculations (Figure S9), the resulting symmetry-reduced W^5+^O_5_ motifs introduce new states slightly below the conduction band minimum (CBM), which effectively lower the bandgap of an idealized 2D nanosheet from 4.17 to 3.64 eV (Figure S9d). While such color centers can lead to a faint blue hue of the otherwise colorless and transparent material (Figure 2d, insert), the ultraviolet–visible (UV–vis) absorbance spectrum (Figures 2d and S10) indicates low absorption in the visible range and strong absorption in the near UV range, consistent with an indirect bandgap of 3.43 eV (Figure S11).^23^ While the small quantity of intrinsic W^5+^ color centers is insufficient to significantly alter the material’s intrinsic color, their presence is thought to be instrumental for the resulting photochromic behavior under UV light exposure, akin to the situation in WO_3_.^29^

We first probe the photochromic behavior of a NbWO_6_ nanosheet suspension under 365 nm UV illumination by adding 10 vol % methanol as a sacrificial electron donor; the relationship between blue color intensity and illumination time is quantified by operando UV–vis spectroscopy (Figure 2e). The NbWO_6_ suspension shows an increase in absorbance intensity in the range of 400–800 nm already after 1 min of 365 nm UV illumination. The absorbance intensity at 575 nm increases significantly up to 20 min illumination, after which a plateau is observed (Figure 2f). We observed different absorption regions within a range of 400–2000 nm in our UV–vis–NIR measurements (Figure S10). The lowest energy absorption peak occurs around 1250 nm (∼1 eV), which is very close to the gap from the calculated Nb polaronic state to the CBM (1.09 eV). The existence of various low-energy absorptions aligns with the versatile polaronic states we identified in the theoretical models.

### Charge Trapping Mechanism

**Figure 3:**
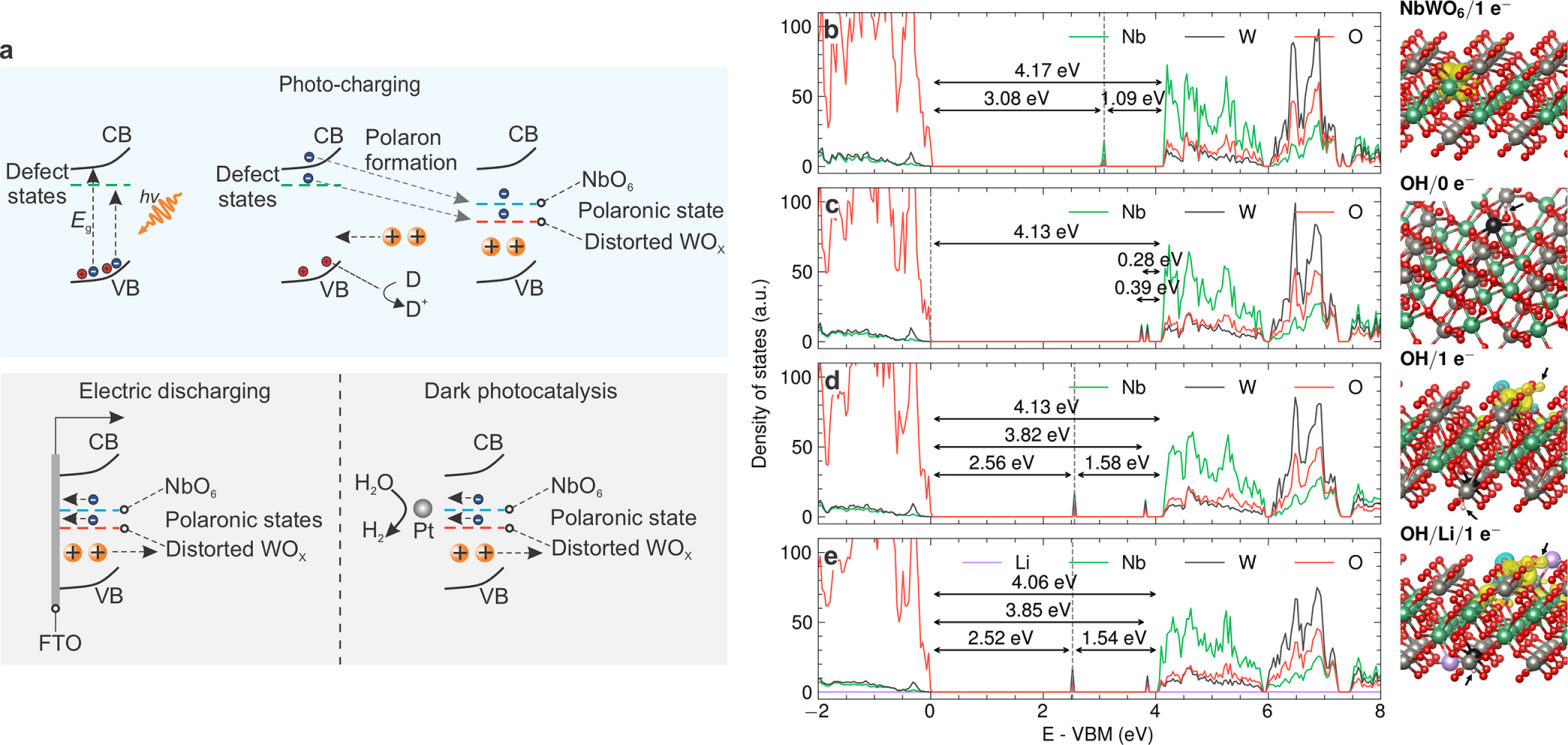
Charge trapping mechanism. (a) Band diagram schematic of the NbWO_6_ photoanode during photocharging, electric discharging and on-demand dark hydrogen evolution processes. The shown process corresponds to photointercalation of cations from the electrolyte. The blue spheres denote electrons, red spheres are holes, and orange spheres are cations that are exchanged with the electrolyte. Partial density of states (PDOS) and spin density plots showing (b) the pristine NbWO_6_ model with a single photoelectron inserted, (c) symmetric hydroxyl model (two hydroxyl groups on opposite sides of the layer) without a photoelectron. To the same model as in (c), but with an additional photoelectron added for (d), which is compensated by an explicit lithium ion in (e). Isosurface level of 0.02 e Å^–3^. The arrows in (c–e), right panel, indicate the hydroxyl group; the dashed line in the band gap separates occupied from unoccupied states. Oxygen, tungsten, lithium and niobium atoms are denoted as red, gray, purple, and green. The distorted tungsten is shown in black. The yellow color in NbWO_6_ model indicates spin density in (b–e).

To explain the observed UV–vis spectroscopic data, we propose the mechanism depicted in Figure 3a, similar to other photochromic metal oxides, such as WO_3_ and MoO_3_. The resulting energy conversion and storage network is described by eqs 1–4.
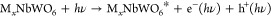
1
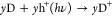
2
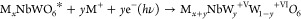
3
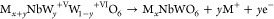
4where M is an electrolyte cation from solution and can include H^+^, Li^+^, and TBA^+^; *D* is the electron donor (here: MeOH) and W in eq 1 can already exhibit some mixed valency if *x* ≠1.

During photocharging, electron (e^–^)–hole (h^+^) pairs are formed upon above-bandgap illumination (eq 1). While the holes migrate to the surface of the material where they are quenched by the electron donor D (eq 2), the photoexcited electrons in the conduction band get trapped at a defect or transition metal site, forming a color center. Under illumination, these in-gap states form the new (quasi-)Fermi level and drive the photointercalation of cations from the electrolyte into the layered host. The concomitant overall increase in the system’s electrochemical potential results in an effective storage of solar energy (eq 3). During the charge releasing phase, the application of a positive current or Pt catalyst extracts the high-energy electrons from the polaronic traps, triggering cation deintercalation (eq 4). This picture is consistent with the double-charge-injection model developed for photochromic tungsten oxide films, which emphasizes the role of both electrons and ions in stabilizing the color centers.^30^

To substantiate the postulated charge trapping mechanism in NbWO_6_, we utilized DFT simulations to examine relevant defects and associated electronic arrangements, which play a pivotal role in the charge trapping performance. In the investigated sample, native exfoliated NbWO_6_ nanosheets possess a negative surface charge compensated by surface-adsorbed TBA^+^ ions and protons. To examine the electronic structure and polaron formation of the sample, which contribute to the stabilization and storage of photoelectrons, a series of single-layer NbWO_6_ models were constructed. In order to model charged species under periodic boundary conditions, a minute positive charge is introduced for each nucleus in the models, ensuring charge neutrality within the system. Detailed information regarding each model, the added nuclear charges, and computational details can be found in the SI Section 2 and Figure S9.

The pristine NbWO_6_ single-layer model exhibits a 4.29 eV bandgap (Figure S9a), with Nb-related conduction bands displaying lower energies (approximately 2.0 eV) compared to those originating from tungsten. Consequently, when an extra electron is introduced into this idealized system, it localizes on one of the Nb atoms located in the central region of the nanosheet, rather than on W, thus forming a polaronic state 1.09 eV below the CBM (Figure 3b). However, in the experimental samples, the WO_6_ motifs in the outer layer contain small amounts of intrinsic oxygen vacancies and tungsten-based color centers, as evidenced by the XPS measurements (Figure 2c). The resulting lattice distortion stabilizes a new state 0.53 eV below the CBM (Figure S9d) which promotes further formation of polarons on W by accommodating photoelectrons. These polaronic states exhibit lower energies than those formed on Nb in the pristine model. In addition, native surface hydroxyl groups can be expected to be present since compensation of the inherent negative charge of the nanosheets occurs either by TBA^+^ or H^+^ as counterions. While bulky TBA^+^ is unlikely to induce noticeable geometric distortions, a specific protonation of an interfacial oxygen will also result in the distortion of the octahedral tungsten environment with concomitant symmetry breaking and a shift of tungsten states to lower energies. In fact, our calculations show that introducing native hydroxyl groups on each side of the symmetric layer model generates new bands, shifted to 0.28–0.39 eV below the CBM (Figure 3c). These initially empty states may as well transform into polaronic states upon the introduction of light-induced electrons into the system. Note that H^+^ may additionally be taken as a proxy for the effect of charge compensation by cations from the electrolyte in stabilizing the W(V) color centers. Since the interaction between surface-bound oxygen and protons is expected to be stronger and more localized than for hydrated Li^+^ ions from the electrolyte, our calculations using protons as charge compensating species represent an upper bound on the energetics for strong polaron–cation interactions while being qualitatively similar to charge screening by alkali metal ions. W–O···H interactions are thus expected to correspond to energetically more stabilized polaron states located at approximately 1.58–1.68 eV below the CBM (Figure 3d), while weaker alkali cation—polaron interactions may lead to more shallow polaron/trap states. This hypothesis is corroborated by pH-dependent photocurrent measurements, which indicate significantly enhanced photocurrent under acidic conditions as compared to alkaline conditions with LiOH as an electrolyte (Figure S12). Importantly, however, due to the layered 2D morphology of the NbWO_6_ nanosheets, both protons and Li ions can efficiently access surface-oxygen sites and form M···O–W (M = H^+^, Li^+^) complexes, compensating the electronic charge on the W(V) color centers. This picture is qualitatively similar to the double charge injection model established for photochromic materials such as WO_3_.^31^

In particular, the environments around native surface O defects allow for further photocharging processes. While the first polaronic state, interpretable as W(V), sits 1.96 eV below the CBM, a second photoelectron may be trapped in a W(IV) state 1.72 eV below the CBM (Figure S9e,g). Note that only one W site neighboring the oxygen vacancy exists in our simplified model and in reality many configurations consisting of spatially close W centers with nearby oxygen vacancies will form a manifold of polaron traps with slightly varying energies for which our calculations present approximate estimates. The second photoelectron may also localize on a Nb in the interior of the multilayered sheet. However, the polaronic state formed by Nb(IV) is less stable than the ones from W(V) and W(IV) by about 0.88 and 0.60 eV, respectively. Overall, the singlet state model (both polarons on W) possesses an energy 0.33 eV lower than the triplet model (one polaron on W and one on Nb), yet all of these trap states can potentially be leveraged to increase the material’s charge storage capacity. The in-gap states also serve as the origin of the observed blue color while according to our calculations a pristine single sheet should be colorless. The various polarons which already form in these simplified models signify the potential for capturing and storing photoelectrons for extended durations. The local distortion induced by hydroxyl groups and oxygen vacancies underlines the potential for tailoring this material to achieve superior performance in terms of augmented capacity and extended storage time, as detailed below.

### Light Harvesting and Photocurrent Generation

**Figure 4:**
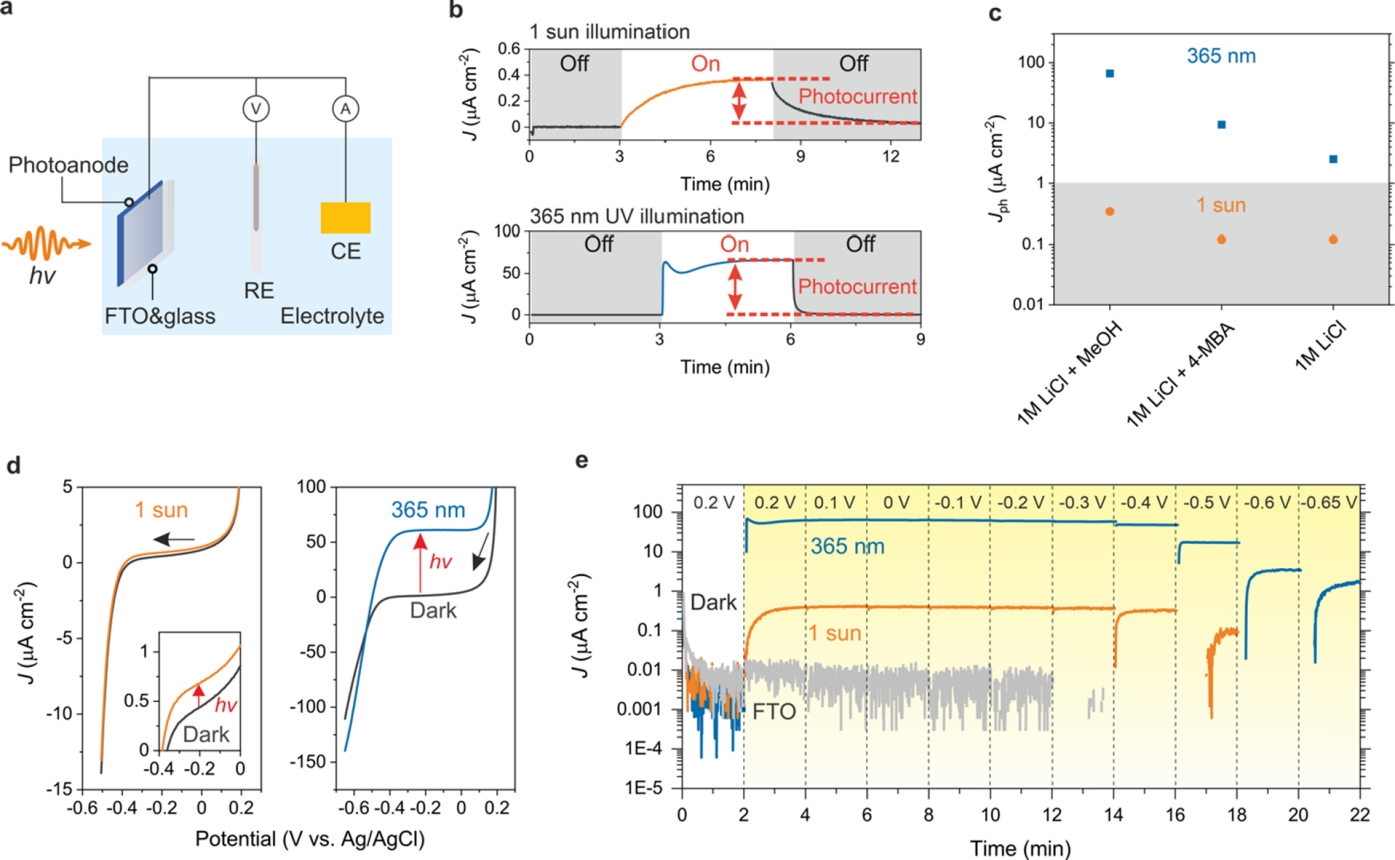
Photoelectrochemical properties of NbWO_6_ photoanode. (a) Schematic illustration of the three-electrode setup for photoelectrochemistry measurements. (b) Photocurrent under (top) 1 sun and (bottom) 365 nm UV illumination. Chronoamperometry experiments were performed with an applied potential of −0.1 V vs Ag/AgCl (saturated KCl). (c) Summary of photocurrents observed in the presence of different electron donors and under different illumination conditions. (d) Linear sweep voltammetry of NbWO_6_ photoanode under 1 sun (left) and 365 nm UV (right) illumination at a scan rate of 10 mV s^–1^. The inset shows the magnified region between 0 and −0.4 V. (e) Photocurrents at different potentials under 1 sun (orange) and 365 nm UV (blue) illumination. Blank FTO substrate under 1 sun illumination is used as reference (gray). The electrolyte used for PEC measurements is oxygen-free 1 M LiCl and MeOH (10 vol %) for b, d, and e.

To investigate the photoelectrochemical (PEC) properties of the NbWO_6_ photoanode, a three-electrode setup with Ag/AgCl (saturated KCl) as reference electrode and Au plate as counter electrode was employed (Figure 4a). The light impinges on the front side of the photoanode to photocharge the material (Figure S13). The resulting photocurrents of the NbWO_6_ photoanode in the presence of different electron donors were measured using chronoamperometry (CA) under dark/light cycles and different illumination conditions. Unless otherwise specified, the light sources used are 1 sun, standard solar illumination (AM 1.5G, 100 mW cm^–2^), and 365 nm UV LED (λ_365 nm_, 206 mW cm^–2^). In all cases, the photocurrent density in the presence of MeOH electron donor is higher than for 4-methylbenzyl alcohol (4-MBA, 10 mM) and H_2_O (Figures 4b and S14–S16), indicating the high efficiency of MeOH for the quenching of holes (Figures 4c, S17, and S18). The transient spike in the photocurrent under UV illumination in Figure 4b may be caused by the discrepancy between photoexcited charge carrier generation, recombination and slow surface reaction dynamics.^32^ The UV–vis absorbance spectra further confirm the higher hole quenching efficiency of MeOH compared to 4-MBA and water, signaled by the more intense blue color of the NbWO_6_ suspension in the presence of MeOH (Figure S19). The color change from light milky to blue is in line with the formation of small polarons as discussed above (Figure 2e and S20).^33^

Note that the presence of oxygen in the electrolyte reduces the photocurrent as oxygen is a potent electron acceptor (Figures S21–S24). Interestingly, however, the photocurrent even reaches 40 μA cm^–2^ under 365 nm UV illumination in spite of the presence of oxygen, which we rationalize with a dynamic equilibrium between electron generation and consumption (Figures S21 and S24). Next, linear sweep voltammetry (LSV) under illumination was conducted to extract the photogenerated electrons (Figure 4d). In both cases, the photocurrent density is higher than the dark current, reaching 61 μA cm^–2^ under 365 nm UV illumination. Scanning toward more negative potentials, the photocurrent slowly decreases until around −0.38 and −0.40 V under 1 sun and 365 nm UV illumination, respectively (Figure 4e). Notably, the photocurrent under 365 nm UV illumination reaches a steady state immediately after turning on the light and is stable for more than 120 min, suggesting efficient and continuous charge carrier generation and extraction (Figure S25).

### Charge Storage Mechanism and Solar Battery Function

**Figure 5:**
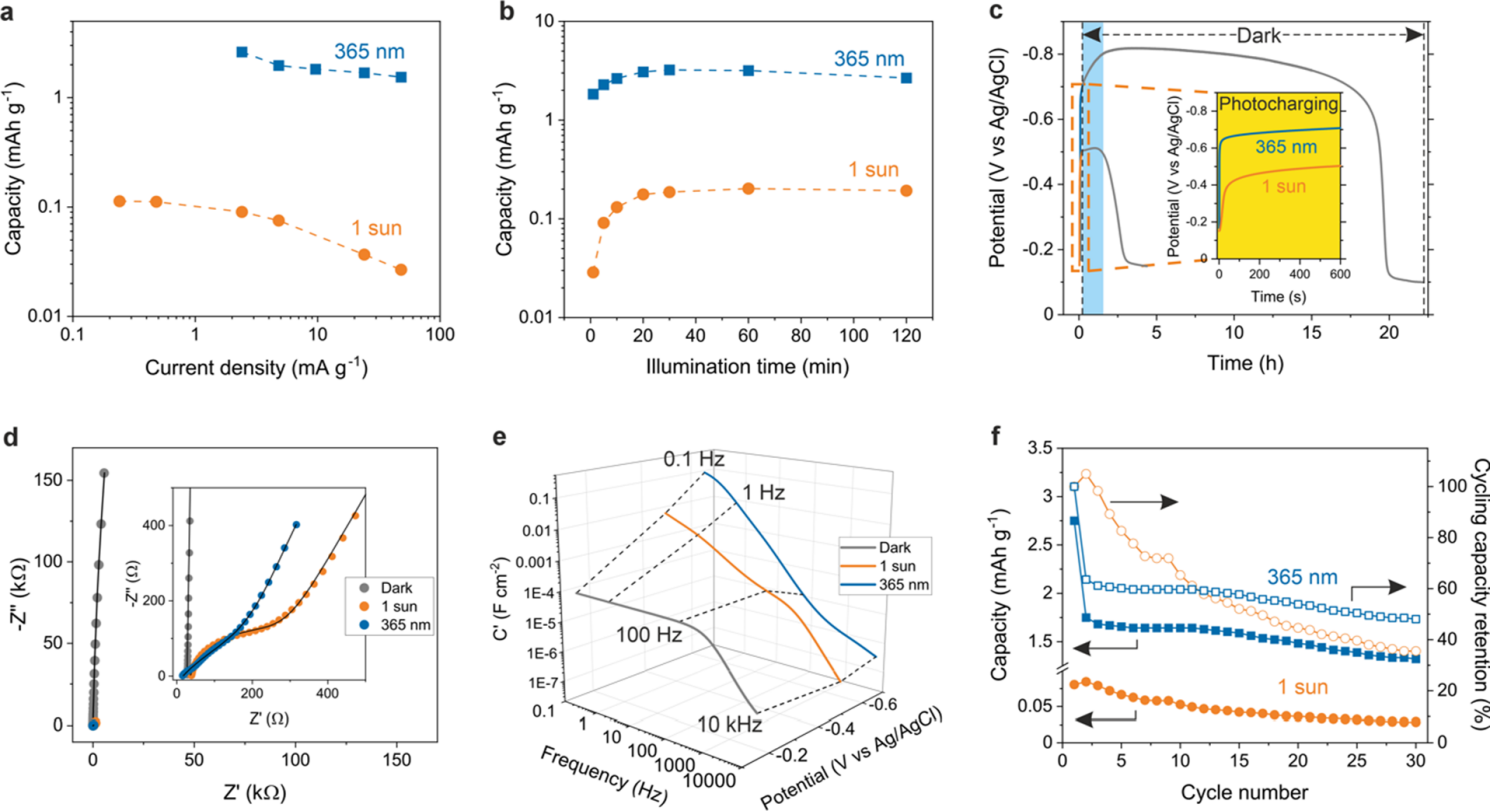
NbWO_6_ charge storage and solar battery half-cell characterizations. The capacity under (a) different discharge current densities after 10 min light illumination, and (b) different illumination times under discharge current densities of 0.48 and 4.8 mA g^–1^ for 1 sun and 365 nm UV, respectively. (c) Open circuit potential (OCP) stability during and after light illumination. The inset shows the OCP during photocharging for 10 min. (d) Nyquist plots under dark (gray) and light illumination. The inset shows the magnified high-frequency region for dark, 360 nm UV and 1 sun plots. (e) The 3D Bode plot of the capacitance vs frequency and potential under dark and light illumination. The dashed lines connect the *C*′ vs potential at a specific frequency. (f) Cycling stability of the photoanode under 1 sun and 365 nm UV illumination, followed by electric discharge. Solid and hollow symbols represent capacity and cycling capacity retention, respectively. The electrolyte used for PEC measurement is oxygen-free 1 M LiCl and MeOH (10 vol %) for (a–e).

First, we study the NbWO_6_ solar battery half-cell performance using a three-electrode setup as shown in Figure 4a to perform photocharging and electric discharging measurements. The photoanode was immersed into oxygen-free 1 M LiCl in the presence of 10 vol % MeOH and a waiting time of around 30 min was applied to achieve equilibration. The photoanode was then photocharged for 10 min under OCP conditions, accompanied by a clear color change to blue (Figures S29 and S30), followed by electric discharge immediately at different current densities (Figures 5a and S31). As expected, the electrode, which was photocharged by 365 nm UV exhibits significantly higher capacity than that illuminated by 1 sun. The considerable reduction in capacity at increased current densities can be attributed to internal resistance. This is likely due to the fact that below the percolation threshold, isolated small polarons on distorted W atoms show low mobilities. To evaluate the maximum photocharging capacity, the photoanode was illuminated for different times, followed by electric discharge (Figure 5b). The OCP increases to about −0.4 and −0.6 V after 1 sun and 365 nm UV illumination for 1 min (Figure S32), respectively, indicating a pronounced photovoltaic effect of NbWO_6_. A further increase in illumination time leads to a slower increase in the photopotential; the OCP reaches around −0.68 and −0.81 V after 1 sun and 365 nm UV illumination for 120 min, respectively. This additional, yet slow increase in photopotential suggests that doubly reduced W(IV) states and Nb(IV) may be additional photocharging channels that can be accessed as the W(V) states are filled, as suggested by the DFT calculations, leading to an additional enhancement in capacity. Subsequently, the photoanode was electrically discharged to 0.2 V. The capacity increases accordingly when increasing the illumination time from 1 to 30 min. Specifically, the capacity reaches 0.18 and 3.2 mA h g^–1^ after 30 min at 1 sun and 365 nm UV illumination, respectively. However, an apparent plateau appears when further increasing the illumination time from 30 to 120 min, indicating a saturation of photogenerated electrons after long-term illumination, which may be due to a steady state of photoexcited electron generation and recombination of charge carriers (Figure 2f). One reason for the low observed capacity (3.2 mA h g^–1^) compared with the theoretical value (71.9 mA h g^–1^) could be the limited electrochemical potential window of water. Second, the number of active storage sites consisting mainly of distorted W sites next to oxygen vacancies is assumed to be limited and to amount to only a small fraction of all W sites residing near the surface. Third, due to the insufficient screening by cations for the bulk W and Nb, only surface W sites get reduced. Lastly, due to the poor electronic conductivity of NbWO_6_ charge transfer is limited, which in turn reduces the accessibility of storage sites, leading to overall low solar conversion efficiency (approximately 0.48%).

Figure 5c shows the OCP stability during and after light illumination. The photopotential reaches around −0.50 and −0.70 V under 1 sun and 365 nm UV illumination for 10 min, respectively. The OCP remains stable for at least 2h and 20h under 1 sun and 365 nm UV illumination, respectively, on average (Figures 5c and S33), suggesting that charge trapping is highly robust in this system. This long-term stability of the sample in the dark after exposure to UV illumination is likely rooted in the atomistic details of the energy storage mechanism, which appears to be the formation of small polarons on the (distorted) tungsten ions. This mechanism gives rise to more stable polarons compared to polaron formation on niobium by roughly 0.52 eV, as dictated by the polaronic state positions in the density of states (DOS) of the pristine and hydroxyl model (Figure 3b,d). Notably, compared to K-PHI, NbWO_6_ exhibits a slower self-discharge rate at a lower discharge current, better rate performance and increased long-term stability of the photogenerated electrons.^1,33^

The population of photoelectrons in the conduction band and associated polaronic/trap states will affect the mobility of the charge carriers, as already observed in related photochargeable materials like 2D K-PHI.^1^ The formation of isolated polarons on the distorted W atoms at short photocharging times may have two consequences: On the one hand, we expect low polaron mobilities and, hence, high charge transfer resistance below the percolation threshold. On the other hand, the limited accessibility of screening cations from solution (H^+^, Li^+^) could help to “de-trap” (i.e., delocalize) the polaron, thus facilitating charge hopping, but also their recombination with photogenerated holes generated in their vicinity. However, as photocharging proceeds and more polarons are formed, intervalence charge transfer by polaron hopping between the different W(IV/V/VI) and Nb(IV/V) sites becomes more facile, which should decrease the charge transfer resistance at longer illumination times.

To identify the influence of light illumination on the charge transfer characteristics and the role of counterions in NbWO_6_, we conducted electrochemical impedance spectroscopy (EIS) in the dark and under light illumination (Figures 5f and S34). Before illumination, the material shows a high resistance *R*_C_ of 7.72 MΩ, assigned to the material’s conductivity (Table S3). Under 1 sun and 365 nm UV illumination, an equivalent circuit composed of a charge transfer resistance (*R*_CT_) in parallel to a constant phase element (CPE1), *R*_C_ in parallel to CPE2, as well as an additional resistor *R*_S_ is used to describe serial resistance including electrode contact and electrolyte (Figure S34 and Table S4). *R*_C_ amounts to around 17.5 kΩ under 1 sun illumination, which is over 2 orders of magnitude lower than in the dark (7.72 MΩ). Notably, *R*_C_ under 365 nm illumination (3.3 kΩ) is decreased by more than 3 orders of magnitude compared to the dark. The *R*_CT_ for the sample under 1 sun and 365 nm UV illumination is less than 300 Ω, suggesting high conductivity of the system under light illumination. The EIS results therefore suggest that above-bandgap illumination significantly decreases *R*_CT_ and *R*_C_. We ascribe the changes in conductivity upon illumination to the generation of small polarons. When reaching a percolation threshold upon continued photocharging, the polaron states can condense into polaron bands, thus leading to a drop in the overall resistance of the sample. A Bode-type plot is further used to understand the energy storage mechanism of NbWO_6_ (Figure 5e). The real capacitance *C*′ exhibits almost constant values in the low frequency range in the dark, indicating a capacitive process. In contrast, *C*′ increases significantly under illumination, indicating a diffusion-limited process.^34^ This latter process is consistent with the photointercalation of cations from the electrolyte between the NbWO_6_ layers or their uptake into the grain boundary regions between the nanosheet aggregates.

Next, the cycling stability of the NbWO_6_ photoanode was investigated by photocharging and electric discharging for 30 cycles (Figures 5f and S35). The photoanodes under 1 sun and 365 nm UV illumination show 35 and 48% cycling capacity retention after 30 cycles, respectively. However, a significant fraction of the capacity reduction in the UV-illuminated electrode manifests after the initial cycle, possibly attributed to an irreversible reaction resulting from water cointercalation, causing the nanosheets to swell and eventually detach, leading to permanent capacity loss.^5^ Comparing with the second cycle as the reference, the photoanode under 365 nm UV illumination exhibits a capacity retention as high as 76% after 30 cycles. This implies that the electrode largely maintains its robustness after the first cycle, akin to the behavior of many battery electrode materials, and the sites involved in polaron formation continue to function efficiently as charge storage reservoirs.

### “Dark” Photocatalysis and On-Demand Hydrogen Evolution

**Figure 6:**
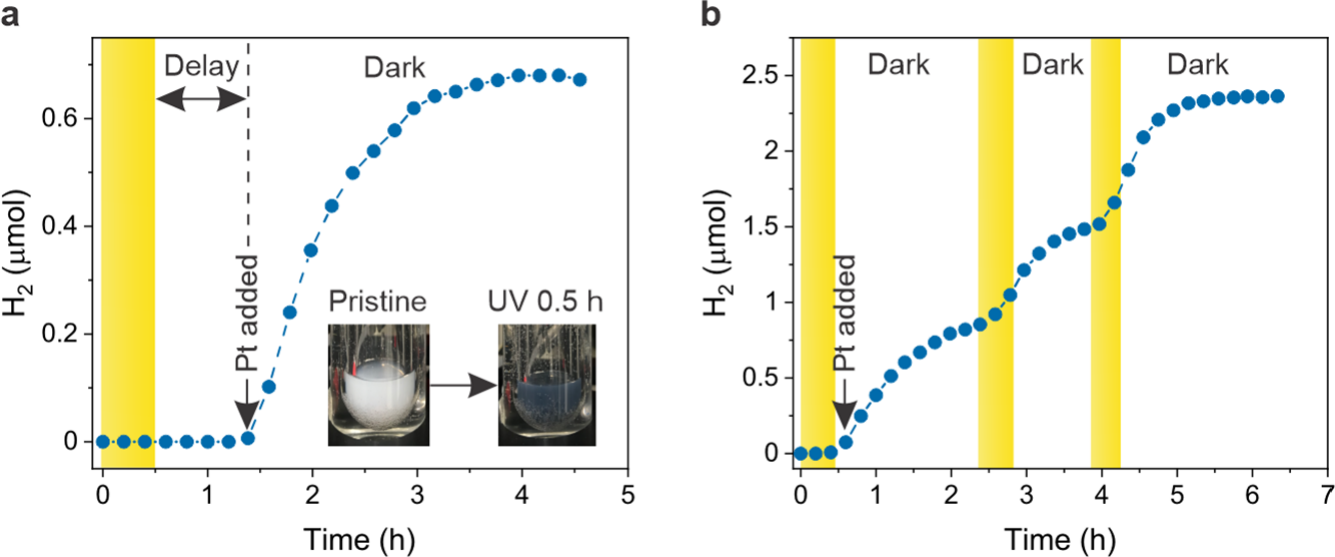
On-demand hydrogen evolution reaction. (a) Dark hydrogen generation as a function of time. The inserts show the color change of the NbWO_6_ suspension from white to dark blue upon 365 nm UV illumination for 30 min. (b) Stability measurement for dark hydrogen evolution. The regions (a, b) highlighted in yellow correspond to the light illumination periods.

Having shown that NbWO_6_ can store photogenerated electrons stably and reversibly qualifies this material as a solar battery photoanode. In order to explore its potential as a dual charge storing and hydrogen evolving material suitable for solar battolyzers, we next assess whether the material’s charge trapping capability can be exploited for dark photocatalysis and on-demand HER. Following the dark photocatalysis scheme established for K-PHI, the photogenerated electrons are trapped and their release is triggered by the addition of a Pt nanoparticle catalyst to generate hydrogen (Figure 3a). Accordingly, a NbWO_6_ (268.3 μmol) suspension was irradiated in the presence of MeOH donor (10 vol %, 24.7 mmol, 92 equiv) under 365 nm UV for 30 min, followed by hydrogen evolution upon the addition of Pt catalyst after 1 h delay in the dark (Figure 6a). The maximum hydrogen generated is 0.68 μmol with maximum turnover frequency (TOF) of 0.13 h^–1^ which corresponds to a 17% loss compared with hydrogen evolution without a delay (max. TOF 0.17 h^–1^) (Figure S36). As expected, photo(dis)charging of NbWO_6_ is accompanied by the material’s color change from white to blue (blue to white) (Figure 6a, insert). Consistent with the time-dependent UV–vis spectrum (Figure 2e), the amount of hydrogen generated saturates after 30 min of illumination, and no significant increase is observed even after 2 h of illumination (Figure S36).

Intriguingly, the 2D NbWO_6_ suspension displays stability after the addition of Pt nanoparticles and consequently enables the on-demand hydrogen generation process multiple times under UV illumination, in spite of the presence of Pt nanoparticles. As shown in Figure 6b, we first illuminated the NbWO_6_ suspension for 30 min, followed by adding the Pt catalyst to produce hydrogen. The hydrogen amount plateaus after around 2 h. We then illuminated the suspension two more times (30 min) without adding more Pt catalyst, followed by a dark phase, during which the hydrogen amount increased again. This behavior is distinct from typical catalysts that evolve hydrogen exclusively under illumination, and point to the possibility of repeated time-delayed hydrogen evolution in the dark phase. One reason for the repeated time-delayed hydrogen evolution in the dark could be a sufficiently high charge transfer barrier between the NbWO_6_ nanosheets and Pt nanoparticles, even in close contact. Once the trap sites are fully occupied during photoexcitation, the charges spillover and continue to migrate to the Pt sites in the dark. The barrier height thus dominates the effective kinetics for charge carrier detrapping in the dark. It should further be noted that protons, rather than Li^+^, likely serve as the main charge compensating species in the aqueous electrolyte during light illumination. Mechanistically, the dark HER can thus be considered as a proton-coupled electron transfer (PCET) reaction with the charged oxide nanosheets serving as both electron and proton donor.^35^ All in all, our experiments suggest that the trapped charges are available for efficient hydrogen evolution upon addition of a catalyst. Further work will explore charge extraction for HER with molecular catalysts which can be separated from NbWO_6_ to regenerate the pristine photoanode for reversible HER and solar battery cycling.

## Conclusions

We have introduced the layered 2D niobium tungstate as a novel optoionic material capable of light harvesting, energy storage, and on-demand conversion into solar fuels. Light storage in NbWO_6_ proceeds via the light-induced formation of polarons residing on the tungsten sublattice, accompanied by hole quenching and photointercalation of Li^+^/H^+^ into the layered host upon above bandgap illumination. Our study thus identifies polaron–cation complex formation as a viable mechanism of charge trapping in transition metal oxides, which may be a more general design concept for novel optoionic materials. Further research is required to enhance light harvesting in the visible range, balance the charge trapping energetics and charge transfer kinetics, and to increase capacity utilization, which is expected to open hitherto unknown avenues to new light storing materials and device designs. Along these lines, the solar battolyzer which combines charge storage (i.e., battery) and solar energy converting (i.e., solar fuel) functions is a powerful concept to mitigate the intermittency of solar irradiation. In addition, a solar battolyzer has the potential to buffer the availability of solar irradiation by working both in light and dark phases, hence resulting in a high degree of utilization: Electricity generated under illumination can be stored in the solar battery component, while excess solar energy is transformed into solar fuels. On the other hand, electricity or solar fuels would be provided upon a shortage in solar energy supply. Identifying materials that can cater to both functions is thus key to leverage the potential of future solar battolyzers. More generally, the combination of light harvesting and charge storage via photointercalation is a versatile next-generation energy concept that mitigates short-term fluctuations of solar energy and opens the door to new optoionic device concepts ranging from photomemory devices to photodesalination.

## Supporting Information Available

Full description of the experimental methods and computational details, powder XRD patterns, and EIS measurement (PDF)
