# Supplementary material for: Decoupling of Light and Dark Reactions in a 2D Niobium Tungstate for Light-Induced Charge Storage and On-Demand Hydrogen Evolution
Source: J Am Chem Soc. 2024 Sep 4;146(37):25467–76. doi: 10.1021/jacs.4c04140 (PMC11421010; doi:10.1021/jacs.4c04140)
Supplement: Supplementary file 1 — ja4c04140_si_001.pdf [file ja4c04140_si_001.pdf]

Supplementary Information

Decoupling of light and dark reactions in a 2D niobium tungstate for light-induced charge storage and on-demand hydrogen evolution

Yang Wang*[1], Yu-Te Chan[2], Takayoshi Oshima[1], Viola Duppel[1], Sebastian Bette[1], Kathrin Küster[1], Andreas Gouder[1], Christoph Scheurer[2,3], Bettina V. Lotsch*[1,4,5]

[1] Max Planck Institute for Solid State Research, 70569 Stuttgart, Germany.

[2] Theory Department, Fritz-Haber-Institut der Max-Planck-Gesellschaft, 14195 Berlin, Germany

[3] IEK-9, Forschungszentrum Jülich, D-52425 Jülich, Germany

[4] Department of Chemistry, Ludwig-Maximilians-Universität (LMU), Butenandtstr. 5-13, 81377 Munich, Germany.

[5] e-conversion, Lichtenbergstr. 4a, 85748 Garching, Germany

* Corresponding authors: yang.wang@fkf.mpg.de; b.lotsch@fkf.mpg.de

1. Experimental section

$NbWO_6$ nanosheets synthesis

Layered α-$LiNbWO_6$ powder was synthesized as reported in the literature.[1] Briefly, Li_2CO_3 (99.999%, Acros), Nb_2O_5 (>99.9%, Roth) and WO_3 (Aldrich) in a molar ratio of 1:1:2 were mixed thoroughly by grinding the mixture for around 15 min. The mixture was calcined at 760 °C for 24 h in the air. The protonated $HNbWO_6 \cdot xH_2O$ was obtained by treating bulk α-$LiNbWO_6$ powder with 0.1 M HCl solution for 3 days and replacing new acid solution every day, followed by washing thoroughly with water and drying at room temperature. The exfoliated $NbWO_6$ nanosheets were obtained by adding an equimolar amount of tetrabutylammonium hydroxide (TBAOH, 40 wt%, Acros) solution into $HNbWO_6 \cdot H_2O$ suspension, followed by stirring for 7 days at room temperature. The suspension was centrifuged at 2 000 rpm for 10 min and collected the top suspension for future experiments.

Photoanode fabrication

The photoanodes were prepared by drop casting 50 µL 5 mg/mL $NbWO_6$ nanosheets suspension on oxygen plasma cleaned fluorine-doped tin oxide glass (FTO, Sigma-Aldrich) substrates with size of 10 × 12 mm^2, and dried on a hot plate at 60 °C for 15 min, followed by annealing at 200 °C in the air for 1 h. To complete the photoanodes fabrication, about 12 mm long copper wire was connected to FTO layer by silver paste. The contact area was sealed with epoxy glue (DP410, 3M Scotch-Weld), leaving an active electrode area of approximately 10 × 10 mm^2. The photoanode thickness is around 850 nm, the mass loading is around 0.20 mg.

Characterizations

Atomic force microscopy (AFM) was performed on Bruker Dimension ICON under Peak Force Tapping mode. The AFM data were analyzed by Gwyddion (version 2.59) software. Scanning electron microscopy (SEM) was conducted on Zeiss Merlin. Transmission Electron Microscopy (TEM) was performed on a Philips CM 30 ST microscope (300 kV, LaB6 cathode). Images were taken with a TVIPS TemCam-F216 CMOS Camera. The program EM-Menu 4.0 Extended was used to perform Fast Fourier Transformations (FFT). Powder X-ray diffraction (PXRD) was conducted on a STOE Stadi P diffractometer (Ag Kα1, Johann-type Ge111 monochromator, triple array of Mythen (Dectris) detectors) in a Debye-Scherrer configuration. The samples were sealed in 0.5 mm borosilicate glass capillaries (Hilgenberg, glass No. 14), which were

spun during the measurements applying a total scan time of 3 hours. Temperature dependent *in situ* PXRD measurements were performed using the same device. A capillary of $HNbWO_6{\cdot}xH_2O$ was heated with a hot air blower (Large Hot Air Gas Blower DGB0001 FMB Oxford). The sample was heated from 25 °C to 300 °C in 25 K steps applying a heating rate of 3K/min. During isothermal hold periods, RXRD patterns were recorded applying a total scan time of one hour and an isothermal delay of 2 minutes prior to every measurement for ensuring thermal equilibration.

X-ray photoelectron spectroscopy (XPS) was conducted on a Kratos Axis Ultra system with monochromated Al Kα x-ray source (hv = 1486.6 eV) under a base pressure better than $5x10^{-5}$ mbar. A charge neutralizer was used to compensate for the sample charging. For binding energy calibration, the main C 1s peak was set to 284.8 eV (adventitious carbon).[2] Casa XPS was used to analyze the data: After subtracting a Shirley background, the peaks were fitted with LA lineshapes, which is a numerical convolution of a Lorentzian and Gaussian function.[3] For the W $4f_{7/2}$ and $W4f_{5/2}$ the binding energy and area ratio were constrained to 2.18 eV and 4:3, respectively. We observed an increase of the W^{5+} amount and the appearance of some Nb^{4+} during longer measurement times. We relate this to some reduction of the $NbWO_6$ probably due to the exposure of the sample to x-rays and low energy electrons by the charge neutralizer. Therefore, the data shown in the main manuscript consist of single Nb 3d and W 4f scans on a fresh sample, which was not exposed to any x-rays or electrons before this measurement started.

Inductively coupled plasma-optical emission spectrometry (ICP-OES) was performed on Varian Vista-PRO (simultaneous ICP-OES spectrometer with axial plasma (Fa. Varian Darmstadt)). The sample was dissolved in HNO_3 (65%), HF (40%) and H_3PO_4 at 165°C for 35min which was diluted with double distilled water. The microwave digestion with Discover SP-D is from CEM GmbH. ICP-Expert software was employed to analyze the data. (In-situ) Ultraviolet-visible (UV-Vis) spectroscopy was performed on Agilent Cary 60 spectrophotometer in transmission and absorption modes. To perform in-situ UV-vis absorption measurement on suspension, the 365 nm UV light was illuminated from the top of a quartz cuvette with a distance of 20 cm. In-situ UV-Vis transmission under different applied bias potentials was conducted by connecting the potentiostat (Autolab PGSTAT302N, Metrohm) with the electrode, which was immersed in

oxygen-free 1 M LiCl electrolyte. Ultraviolet-Visible-Near Infrared (UV-Vis-NIR) spectra was conducted on Cary 5000 UV-Vis -NIR (Agilent Technologies).

PEC measurement

All (photo)electrochemistry measurements were performed in a home-made closed glass reactor equipped with a quartz window for light illumination. An Ag/AgCl electrode (saturated KCl, RE-1CP) was used as reference electrode and an Au foil was used as counter electrode. Unless otherwise specified, all potentials in this work were measured versus Ag/AgCl. Oxygen-free 1M LiCl (Roth) aqueous solution was used as electrolyte. Methanol, 4-methylbenzyl alcohol (4-MBA) and water were used as donor. The electrolyte was purged with Ar for at least 30 min prior to every measurement through a porous glass frit to remove dissolved oxygen. Artificial sunlight was provided by a Sciencetech LightLine A4 solar simulator (class AAA) fitting the ASTM standard G138 (AM 1.5G). The 365 nm UV illumination was provided by Thorlabs M365LP1-C4 lamp at an operating current of 1700 mA. The sunlight intensity was measured by a calibrated Thorlabs S130C/PM100D thermal power meter. The light intensity for 365 nm UV was measured by a calibrated Thorlabs S120VC standard photodiode power sensor and PM100D Thorlabs power meter. The solar-to-output efficiency was calculated from photocharging and electric discharging measurements. The theoretical capacity is calculated by:

$$Q_{\mathrm{theoretical}} = \frac{nF}{3.6m} = \frac{1 \times 96{,}485.3\ \mathrm{C\ mol^{-1}}}{3.6\ C\ \mathrm{mA^{-1}h^{-1}} \times\ 372.74\ \mathrm{g\ mol^{-1}}} = 71.9\ \mathrm{mAh\ g^{-1}}$$

Where *n* is the number of electrons transferred per formula unit, we only count W here. *F* is Faraday’s constant, 3.6 is a conversion factor between coulombs and mAh, and *m* is the molar mass per formula unit.

(Photo)electrochemical measurements were performed on a multichannel potentiostat (Autolab M204, Metrohm). The dark electrochemical impedance spectroscopy (EIS) was performed under different applied bias potentials from 10 kHz to 0.1 Hz. The light EIS was conducted under 340 nm UV LED and 1 sun irradiation for different time from 10 kHz to 0.1 Hz.

The real capacitance C’ is defined as[4]:

$$C' = \frac{-Z''}{\omega|Z|^2}$$

Where –Z’’ is the imaginary impedance, ω is the frequency, Z is the electrochemical impedance.

Dark photocatalysis

The dark hydrogen evolution experiments were conducted in a home-made glass reactor with quartz window on top for illumination and thermostated at 25 °C as previous described[5,6]. The reactor was soaked in aqua regia overnight before dark photocatalysis experiment. The 365 nm UV illumination was provided by Thorlabs M365LP1-C4 lamp at an operating current of 1700 mA. The $NbWO_6$ nanosheets (95 mg) were dispersed in water (8 mL) with the presence of MeOH (1 mL). The headspace of the reactor was evacuated and argon backfilled several times to remove the air. The suspension was stirred at 400 rpm during illumination. For the dark hydrogen evolution experiments, the platinum nanoparticles catalyst (1 mL of 1000 ppm aqueous colloidal solution, Aldrich) was injected into the photocharged suspension with different delay time. To measure the amount of hydrogen evolution upon adding the Pt catalyst, the headspace of the reactor was periodically sampled and the amount of evolved gases was quantified by gas chromatography (Shimadzu GC-2030). This gas chromatograph is equipped with a Barrier Discharge Ionization detector (BID) and Thermal conductivity detector (TCD) using argon as the carrier gas. Unless stated differently, evolution of gases in the headspace is measured in a closed system (i.e., batch measurements). Control experiments confirmed that no hydrogen was generated in the absence of $NbWO_6$, light or Pt catalyst.

2. Computational details

We conducted electronic structure calculations utilizing the FHI-aims program package. All structural optimizations employed the HSE06 hybrid functional to ensure precise electronic structures. Owing to the size of the models, numerical convergence was attained with a light basis set. The reciprocal space was sampled through a $2 \times 2 \times 1$ Monkhorst-Pack k-point grid. The structures were utterly relaxed until the forces fell below $5 \times 10^{-2}\ eV/Å$.

To identify potential polaron formation sites, we initially executed relaxation by applying PBE+U and implementing a Hubbard U correction on a single atom of the chosen element. In this particular case, we determined that $U = 1.2$ was sufficient for pre-relaxing the structure towards the desired polaron formation, using a tight basis set. Subsequently, the pre-relaxed

structure was subjected to a comprehensive relaxation protocol at the HSE06 level without the +U correction, yielding the final geometry and electronic structure.

Models

The single-layer $Li\mathrm{NbWO}_6^{\square}$ / NbWO_6^- structures were derived from the pristine $LiNbWO_6$ structure obtained from the Materials Project. [10.17188/1278002] The two-hydroxyl model was constructed by incorporating two hydrogen atoms symmetrically onto oxygen atoms in the single layer. The oxygen vacancy model was generated by eliminating one oxygen atom bound to one of the tungsten atoms.

Virtual crystal approach

In order to model the negatively charged single-layer NbWO_6^- system under periodic boundary conditions, we employed the virtual crystal approach (VCA) by adjusting the charge of atomic nuclei. A minuscule extra charge q^{extra} was incorporated into each nucleus. The q^{extra} value is system-dependent and conforms to $Eq.1$. The compensating charge q^{extra} is primarily governed by three factors: 1. numbers of missing cations n^{cat}, 2. numbers of introduced photoelectrons n^{pe}, 3. numbers of atoms sharing the counterbalance charge n^{atoms}. The missing cations arise from the exfoliation process, during which excess $TBAOH$ reacts with $H\mathrm{NbWO}_6^{\square}$ and, theoretically, yields TBA^+ and NbWO_6^-. For instance, the two-hydroxyl model with zero photoelectron has chemical formula $H_2Nb_{32}W_{32}O_{192}^{-30}$. There are 30 missing cations and photoelectrons and 258 atoms for sharing counterbalance charge, resulting in a q^{extra} equal to 0.1162. Since the NbWO_6^- single layer is isolated by bulky TBA^+ species, we only modeled the negatively charged single layer within the simulation box. The models and their corresponding additional q^{extra} values are presented in Table S1.

$$q^{extra} = \frac{n^{cat} + n^{pe}}{n^{atoms}} \qquad (Eq.1)$$

$$q^{extra} = \frac{n^{cat}+n^{pe}}{n^{atoms}} \qquad (Eq.2)$$

Table 1. The added nuclear charge, q^{extra}, for each model.

Models	$\mathrm{LiNbWO}_6^{\square}$	NbWO_6^-	$\mathrm{NbWO}_6^- - Ov$	$\mathrm{NbWO}_6^- - 2OH$

# of photoelectrons				
0	0	0.1250	0.1176	0.1162
1	0.0035	0.1289	0.1216	0.1202
2			0.1255	0.1240

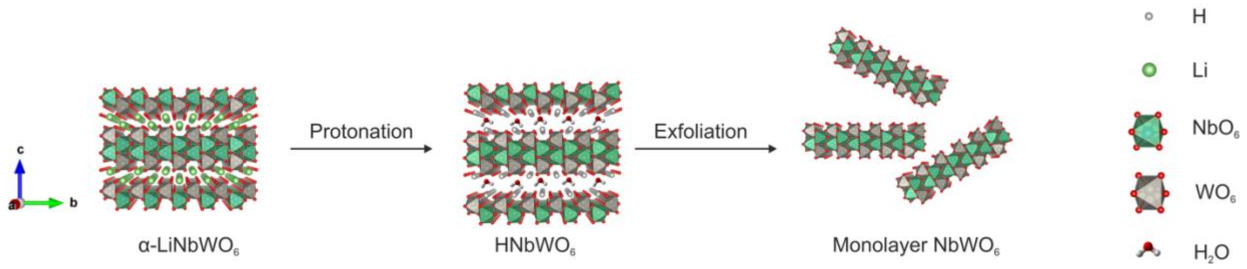

Figure S1. Schematic illustration of the liquid phase exfoliation process for 2D $NbWO_6$ nanosheets.

XRPD data analyses and crystal structure refinement

The program TOPAS 6.0 (ref. 7) was used to refine the recorded XRPD data. The peak profile was described by the fundamental parameter approach implemented into TOPAS (ref. 8) and the background modeled by Chebychev polynomials of 6th order. The pattern of $LiNbWO_6$ was subjected to a fully weighted Rietveld refinement[9] using the dataset of α-$LiNbWO_6$ published by Fourquet et al.[1] as starting model. In the crystal structure of α-$LiNbWO_6$ there are three metal position situated all on 2*c* sites. In their crystal structure refinement Fourquet et al. used an ordered distribution of the cations within the cation substructure, i.e. lithium, tungsten and niobium occupying separate sites (Table S2, second column). However, they point out the possibility of a cation disorder, which they could not rule out due to the limited quality of the sample and the X-ray and neutron scatting data. Using a structure model of α-$LiNbWO_6$ with ordered cations only led to a very poor fit of our diffraction data (Fig. S2a). An inspection of the Fourier map revealed considerable positive residual electron density at the lithium site (=metal(1)), slight positive residual electron density at the niobium sites (= metal(2)) and considerable negative residual electron density at the tungsten site (= metal(3)) indicating occupational disorder among the cations. In the first attempt we tried to model the disordered cation substructure isotropically, i.e. the lithium site is partially substituted by equivalent amounts of tungsten and niobium, the niobium site is partially substituted by equivalent amounts of tungsten and lithium and so on (Table S2, third column). The led to a significant improvement in the refinement (Fig. S2b) and yielded an acceptable R-wp value (4.03 %). However, there is still some misfit and this model does not properly account for what was observed in the residual electron density map. In X-ray diffraction the scattering power roughly scales with the number of electrons. For the cations we consider: Li^{+} = 2 electrons, Nb^{5+} = 36 electrons and W_{6+} = 68 electrons. The significant positive residual electron density observed for the lithium position in the refinement using a model with ordered cations, can be explained by the presence of niobium and/or tungsten on this position. The negative residual electron density observed for the tungsten position can be explained by the presence of niobium and/or lithium on this position, with lithium having the stronger impact. For the niobium position a slightly positive residual electron density was observed. If niobium cations (36 electrons) were substituted isotropically by lithium and tungsten cations ((2 electrons + 68 electrons)/2 = 35 electrons) this would yield in hardly detectable negative residual electron

density. Only an excess of tungsten can lead to a slightly positive residual electron density. Hence, the presence of an anisotropically disordered cation substructure appears to be more suitable. For testing this, we refined a structure model in which lithium and niobium are partially replaced by tungsten and tungsten is partially replaced by both lithium and niobium (Table S2, fourth column). This led to an additional improvement of the fit (Fig. S2c) and a lower R-wp value (3.74 %). Since we cannot determine the distribution of the cations among the metal positions and as the refined atomic coordinates do not differ significantly from the dataset published by Fourquet et al.[1] we did not deposit a new crystal structure dataset into the databases.

Table S2. Occupancies of the cation positions used in the crystal structure model of α-$LiNbWO_6$ in the refinement of the PXRD data of the $LiNbWO_6$ sample.

site<br>(all 2*c* sites)	ordered cations	isotropically<br>disordered cations	anisotropically<br>disordered cations
metal(1) @ 0, 0.5, 0.42	1 Li	1-2x Li<br>x Nb<br>x W	1-x Li<br>x W
metal(2) @ 0, 0.5, 0.09	1 Nb	x Li<br>1-2x Nb<br>x W	1-y Nb<br>y W
metal(3) @ 0, 0.5, 0.73	1 W	x Li<br>x Nb<br>1-2x W	x Li<br>y Nb<br>1-x-y W
refined parameters	-	x = 0.122(2)	x = 0.148(4)<br>y = 0.107(6)

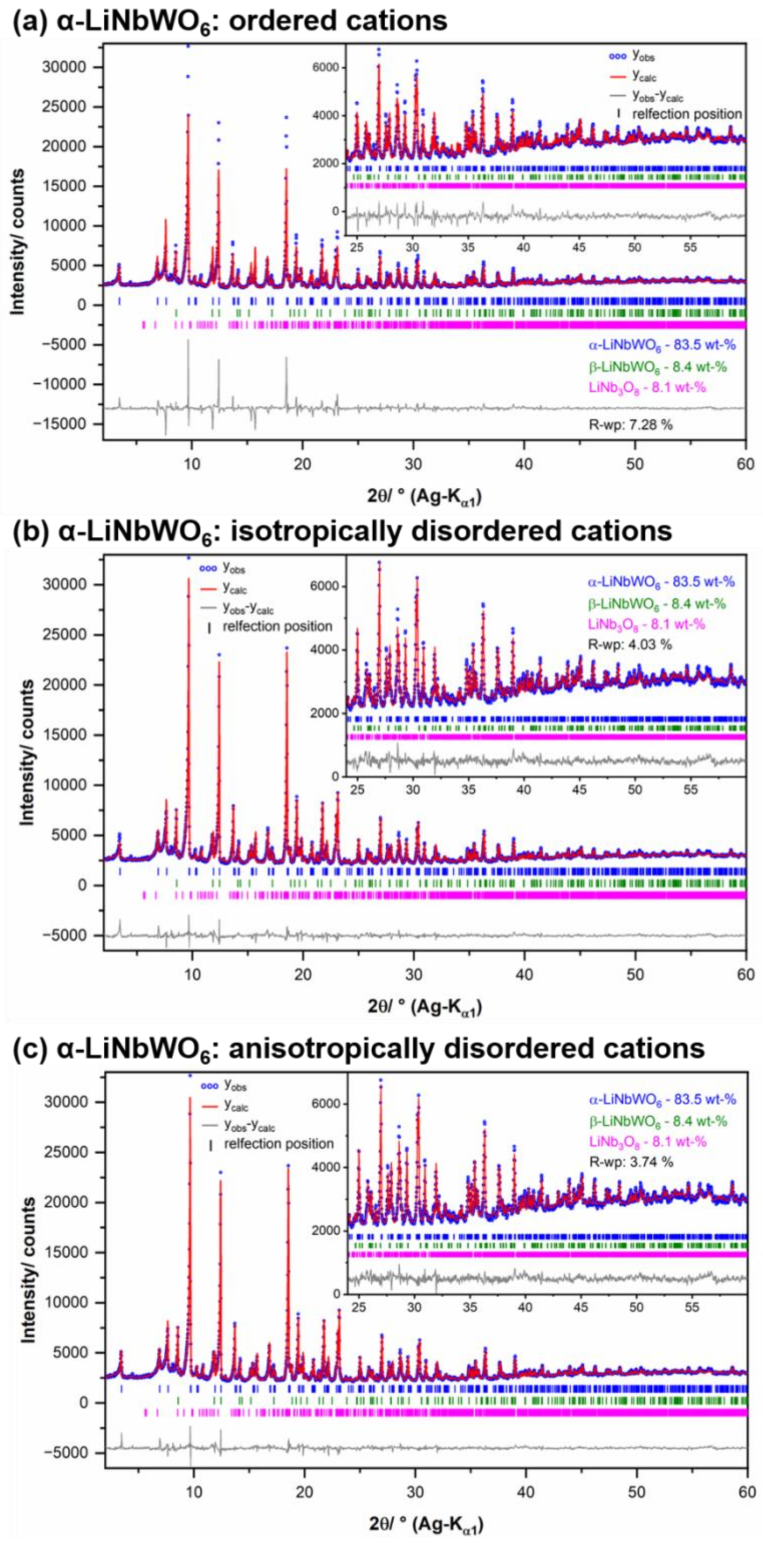

Figure S2. Graphical results of the final Rietveld refinements of the XRPD pattern of α-$LiNbWO_6$ using different structure models in terms of cation ordering.

The XRPD analyses of the $LiNbWO_6$ sample showed that the powder mainly consist of α-$LiNbWO_6$ (83.5 wt-%) with minor impurities of β-$LiNbWO_6$ (8.4 wt-%) and $LiNb_3O_8$ (8.1 wt-%) (Fig. S2c, Fig. S3, green and magenta tick marks). After protonation the diffraction pattern

fundamentally changes (Fig. S3) but reflections attributed to the impurities are not effected indicating that neither in β-$LiNbWO_6$ nor in $LiNb_3O_8$ lithium ions are exchanged by protons. By protonation, the 001 peak of α-$LiNbWO_6$ shifts towards lower diffraction angles corresponding to an increase in the lattice plane distance from 9.27 Å to 13.01 Å and increases in intensity. This is attributed to the intercalation of water molecules into the structure forming an $HNbWO_6 \cdot xH_2O$ type compound. The slight downshift of non-00l reflections also indicates that the lateral dimension of the layers slightly expanded upon protonation and water intercalation. A LeBail fit[10] of the pattern using space $P\overline{4}2_1m$ and *a*- and *c*- lattice parameters adapted to the peak shift failed. As neither reducing the space group symmetry to $P\overline{4}$ as well as reducing the lattice symmetry yielded a suitable fit, we believe that the protonation leads to a fundamental change in the unit cell metrics. As many reflections seem to be unaffected by protonation (at d ≈ 3.3 Å, 2.4 Å, 1.7 Å and 1.5 Å, Fig. S3), we believe that the setup of the layers is not undergoing a fundamental change during the transition from $LiNbWO_6$ to $HNbWO_6 \cdot xH_2O$. Due to the pronounced anisotropic peak broadening, which is indicative for structural disorder the powder patterns cannot be indexed. We think that the structural disorder originates from the positional disorder in the cation substructure. As in β-$LiNbWO_6$ in which all metal cations are occupationally disordered lithium cations cannot be exchanged by protons, we believe that the proton exchange is incomplete in the $LiNbWO_6$. An intergrowth of proton-exchange and non-exchanged layers in the material may lead to pronounced structural disorder.

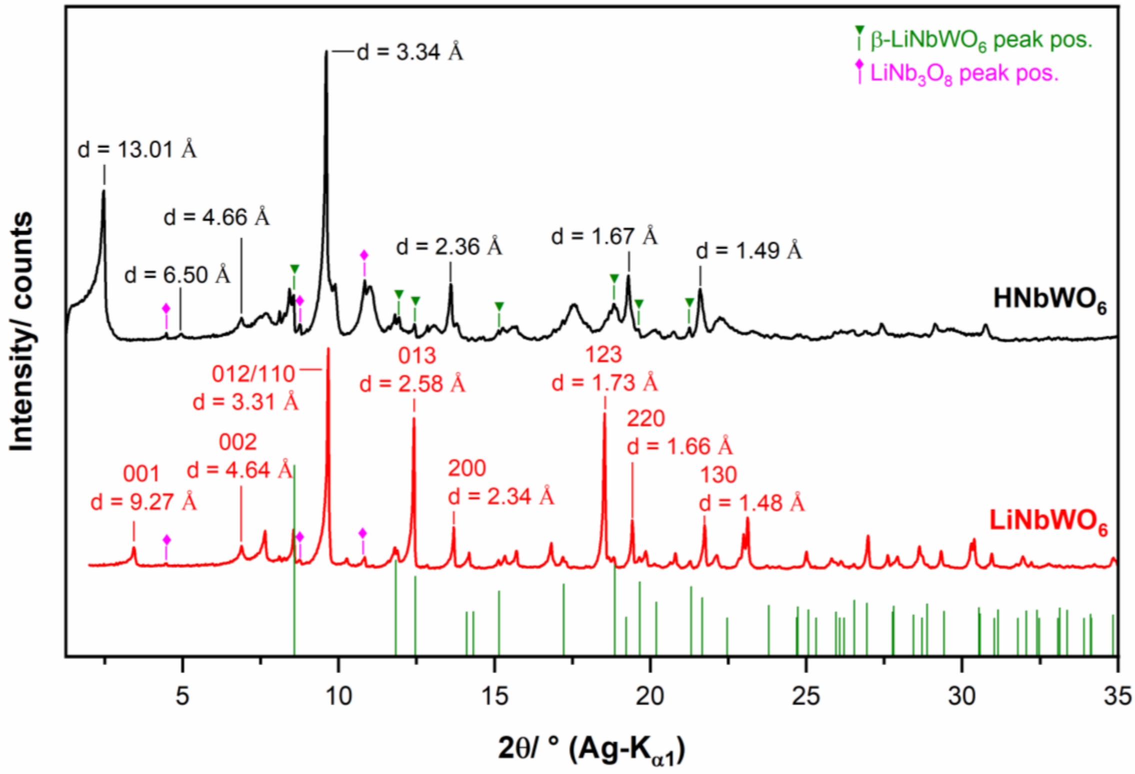

Figure S3. The powder XRD patterns of $LiNbWO_6$ and protonated $HNbWO_6{\cdot}xH_2O$ powder including selected reflections indices and corresponding d-spacings for α-$LiNbWO_6$ in the $LiNbWO_6$ sample (red font color), selected d-spacings of diffraction lines in the $LiNbWO_6$ sample (black font color) and peak positions of β-$LiNbWO_6$ (green) and $LiNb_3O_8$ (magenta) impurities.

Temperature dependent *in situ* PXRD measurements (Fig. S4) give insights into the $HNbWO_6{\cdot}xH_2O$ material. Moderate heating at 75 °C leads to a significant change in the diffraction pattern (Fig. S4a, i and ii). The basal reflection corresponding to a d-spacing of 13.01 Å is shifted towards 10.7 Å (Fig. S4b), i.e. the interlayer spacing is significantly contracted. This points to the presence of loosely bound water molecules in-between the layers. In addition, broad peaks situated at 7.8 and 11.2 ° 2θ disappear indicating a reduction of structural disorder by the release of water molecules. Further heating above 150 °C leads to a gradual broadening of the basal reflection, which eventually disappears, whereas all other peaks remain sharp. This is most likely attributed to either the gradual release of additional water molecules or the dehydration of hydroxide groups, which leads to an increasing modulation of the interlayer distance and at the end of the process the material loses its layered character. Neither the reflections of hydrated $HNbWO_6$ nor of dehydrated $HNbWO_6$ can be assigned to any known compound. Peaks attributed to the minor impurities

LiNbWO$_6$ and LiNb$_3$O$_8$ (Fig. 4b, magenta and green tick marks) can be observed throughout the entire temperature range.

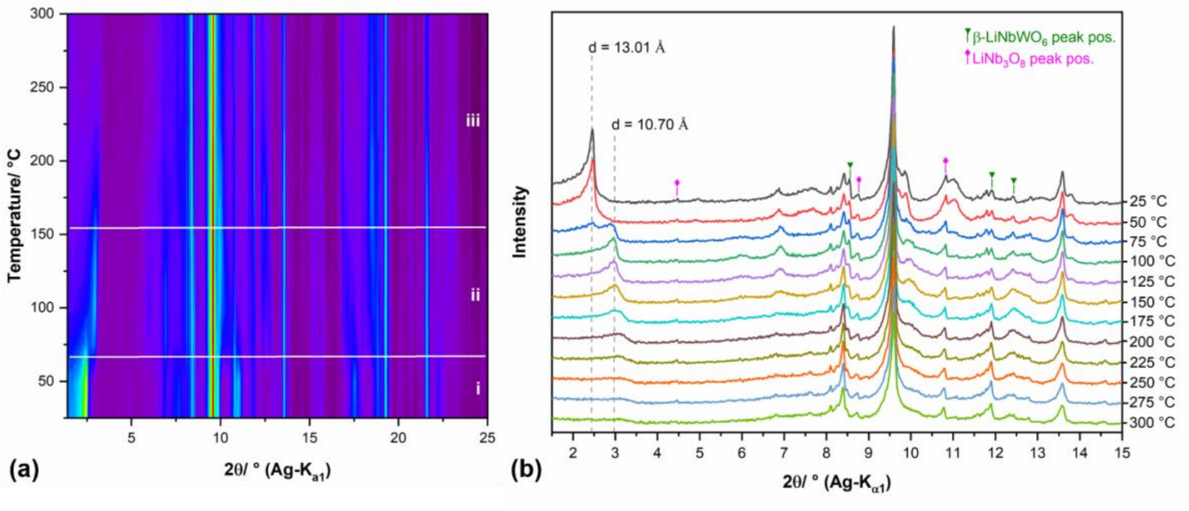

Figure S4. (a) Temperature dependent *in situ* PXRD patterns of the HNbWO$_6$ sample (HNbWO$_6$·xH$_2$O type compound). White lines indicate phase transitions (from i to ii and subsequently to iii). (b) excerpt of the temperature dependent *in situ* PXRD patterns of the HNbWO$_6$ sample including d-spacings of selected reflections and peak positions of β-LiNbWO$_6$ (green) and LiNb$_3$O$_8$ (magenta) impurities.

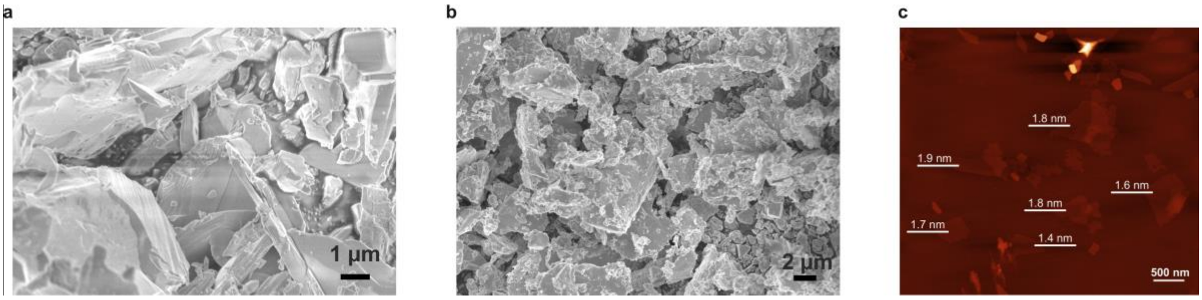

Figure S5. SEM images of (a) $LiNbWO_6$ and (b) $HNbWO_6{\cdot}xH_2O$ powders, which exhibit well-defined layered structures. (c) AFM image of exfoliated 2D $NbWO_6$ nanosheets which were exfoliated from $HNbWO_6{\cdot}xH_2O$ with the presence of TBAOH as shown in Fig. S1. The possible counter ion is TBA^+ to compensate the negative charge of the nanosheets.

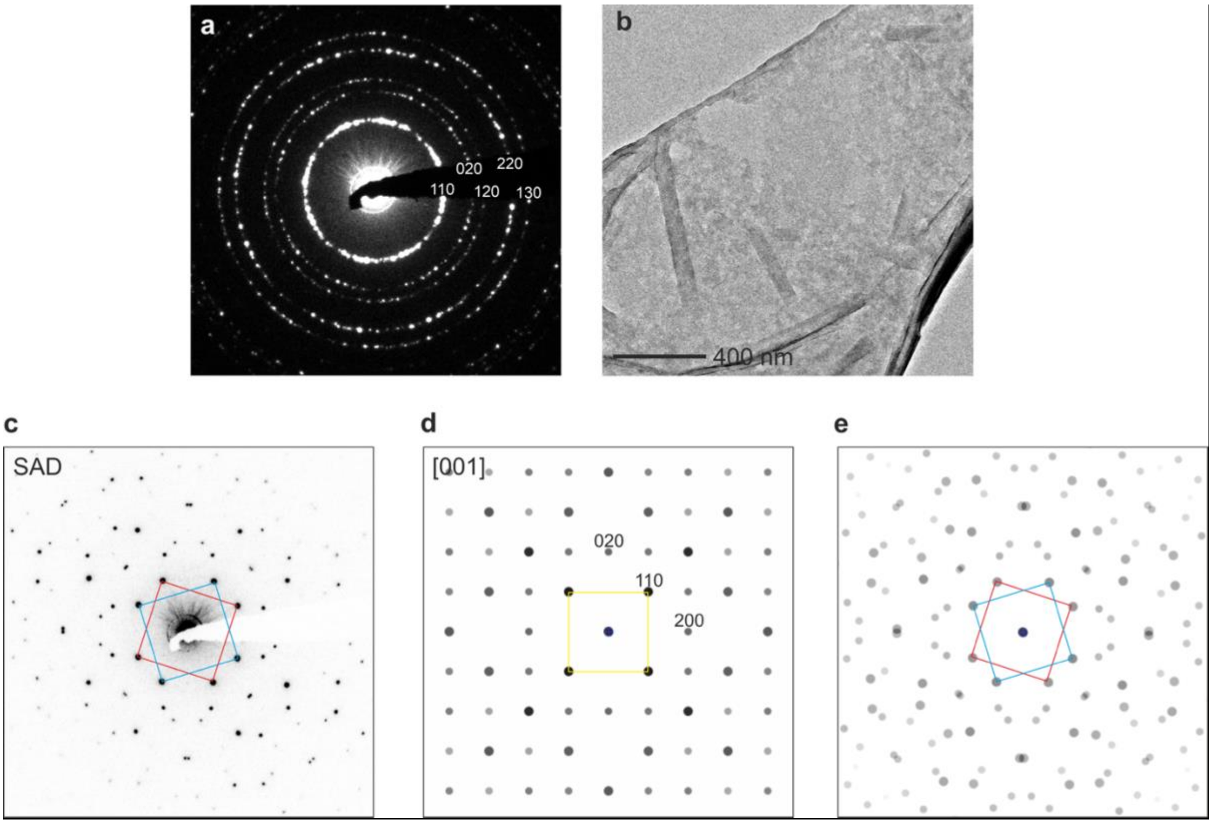

Figure S6. (a) The (*hk*0) selected-area electron diffraction (SAED) pattern from a multilayer array of $NbWO_6$ nanosheets, indicating the presence of crystalline yet turbostratically disordered few-layer nanosheets, which is consistent with the facile exfoliation and restacking of the layered solid into single-layer nanosheets with rotational layer offsets. (b) TEM image of 2D $NbWO_6$ restacked on a TEM grid. (c) The experimental SAED pattern of two layer NbWO6 sheets. The simulated patterns of (d) single layer and (e) two layers sheets with rotation from bulk $LiNbWO_6$.

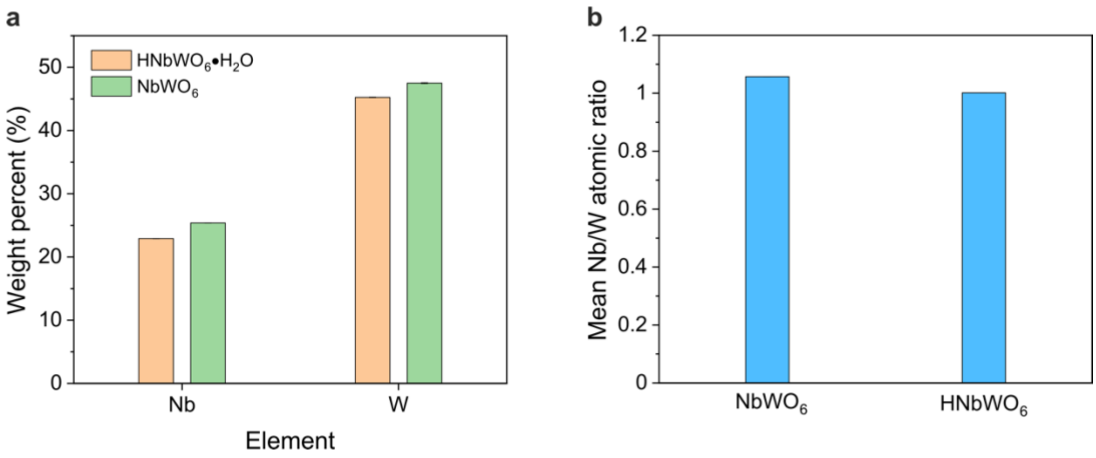

Figure S7. (a) The analytical results from ICP-OES on the weight percent of Nb and W in $HNbWO_6{\cdot}xH_2O$ and $NbWO_6$ powder. (b) The calculated molar ratios between Nb and W are 1.00 and 1.06 for $HNbWO_6{\cdot}xH_2O$ and $NbWO_6$, respectively. Values are means with standard deviation from two different measurements.

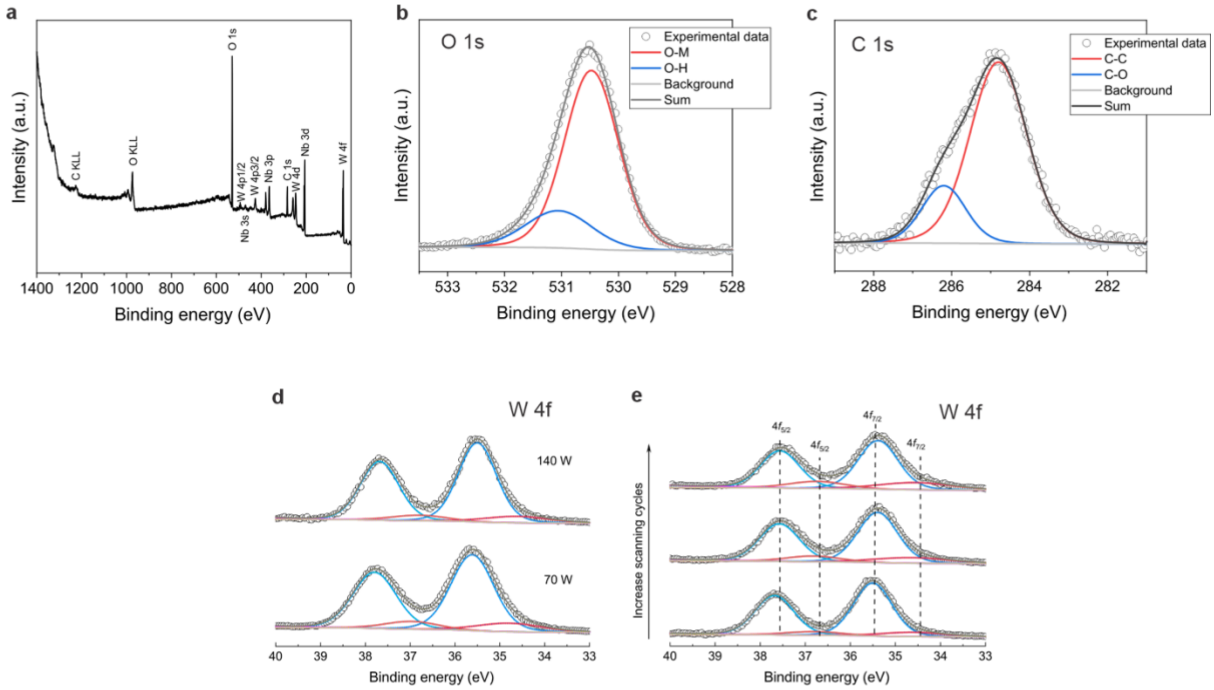

Figure S8. (a) XPS survey scan spectrum of NbWO6. High resolution XPS of (b) O 1s, (c) C 1s, (d) W 4f spectrum under different X-ray powers, and (e) W 4f spectra development with scanning time under the power of 140 W in $NbWO_6$. The increase in the red line shows the increasing fraction of W(V).

The XPS survey (Fig. S8a) indicates Nb, W and O as well as some C impurities. In Fig. S8b the O 1s spectrum is displayed, which shows a second component around 531.4 eV besides that attributed to the O-W and O-Nb bonds around 530.5 eV, which we relate to the OH components. XPS data of W 4f taken under different power and charge neutralizer settings (Fig. S8d), which are 70 W, 1.2 A, 1.5 V and 140 W, 2A, 3.5 V, do not show significantly different W(VI): W(V) ratios, which are around 7:1 and 6:1, respectively. However, we observed an increase in the fraction of W(V) over the scanning time (Fig. S8e). The last spectrum in Fig. 8e was taken approx. 2 h 15 min after the first one. Therefore, we used the first scanning data, which we approximate to the "pristine state" in the manuscript.

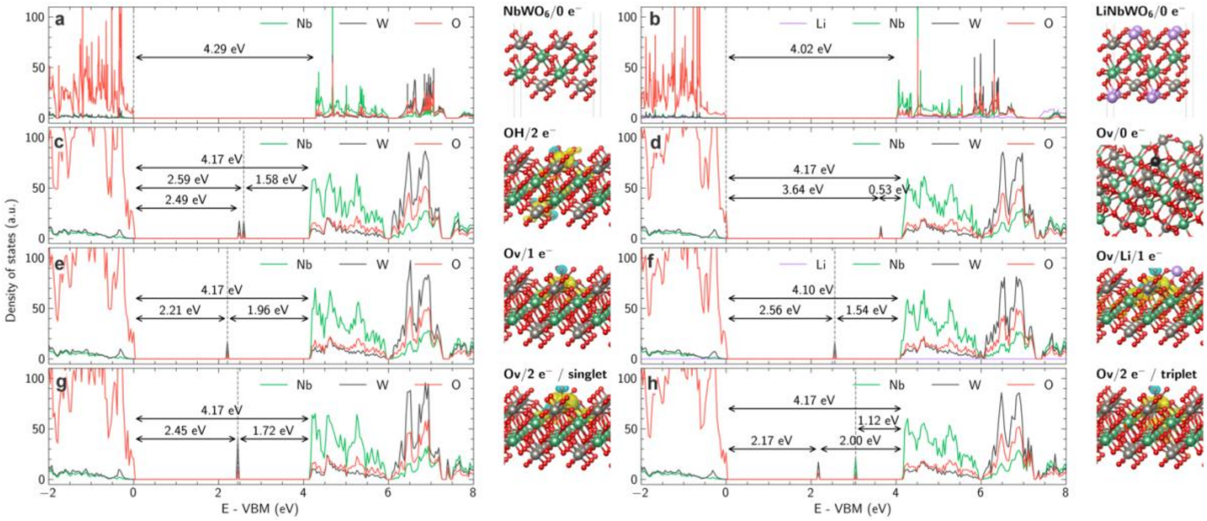

Figure S9. PDOS and spin density plots (a) pristine $NbWO_6$ models with zero photoelectron. (b) Pristine LiNbWO6 model without photoelectron. (c) Two-hydroxyl model with 2 photoelectrons. Oxygen-vacancy models with (d) zero photoelectron, (e) one photoelectron, (f) one electron with one Li, (g) two (singlet) photoelectrons and (h) two (triplet) photoelectrons. Isosurface level of $0.02\ e\ \text{Å}^{-3}$. Oxygen, tungsten, niobium and lithium atoms are denoted as red, grey, green and purple, respectively. The distorted tungsten is denoted as black.

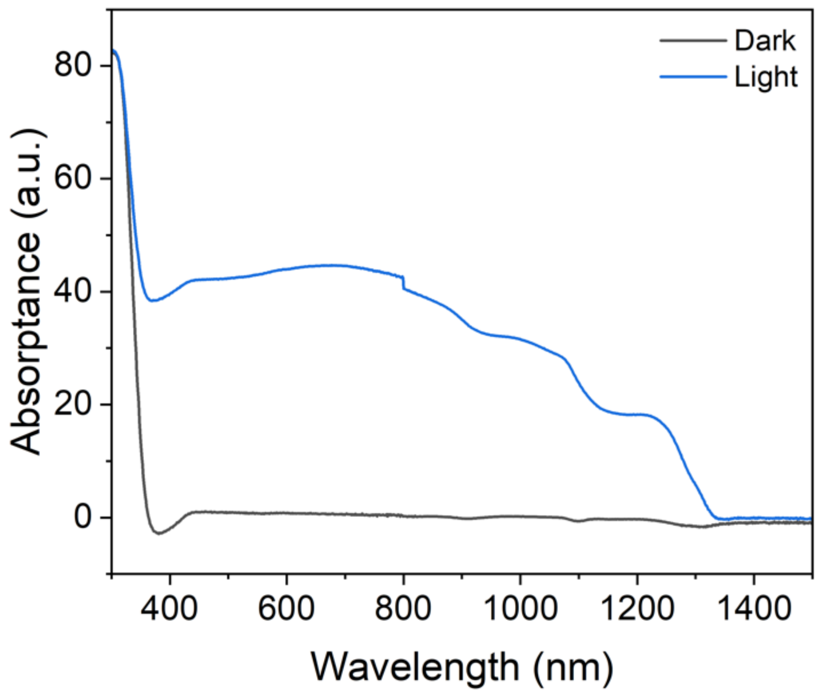

Figure S10. The UV-Vis-NIR spectra of 1 mg ml^{-1} $NbWO_6$ suspension in the presence of 10 vol% MeOH before and after 365 nm UV irradiation for 30 min.

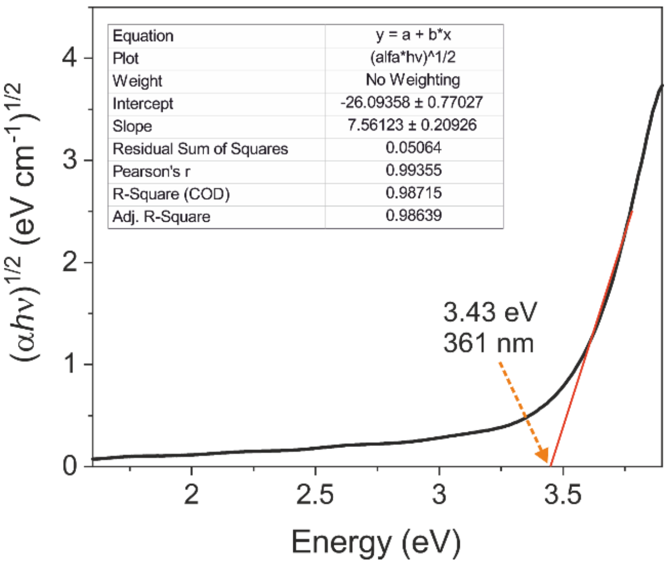

Figure S11. The Tauc plot of $NbWO_6$ nanosheets obtained from UV-vis indicates an indirect band gap of 3.43 eV.

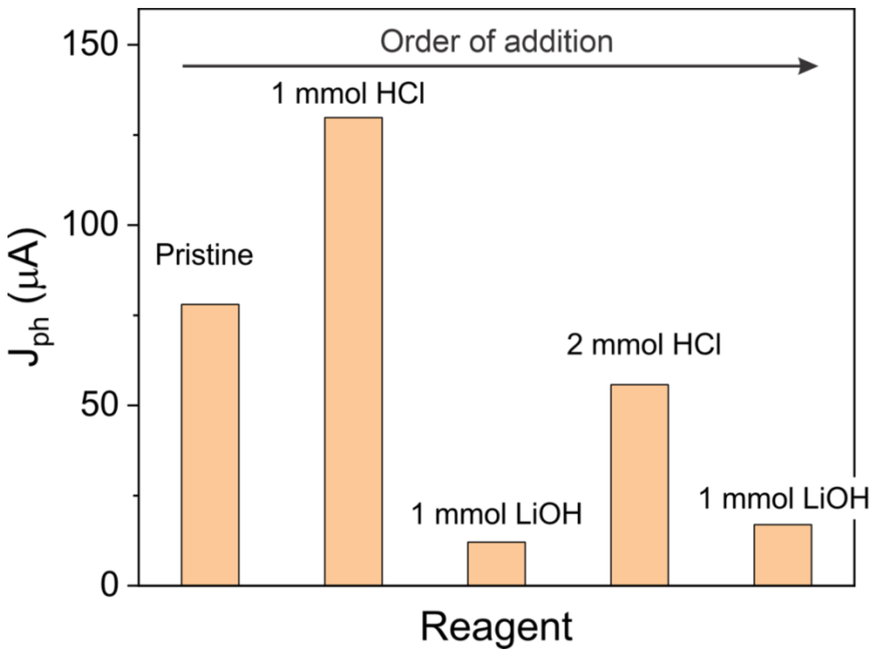

Figure S12. pH-dependent photocurrent in oxygen-free 1M LiCl in the presence of MeOH donor under 365 nm UV illumination.

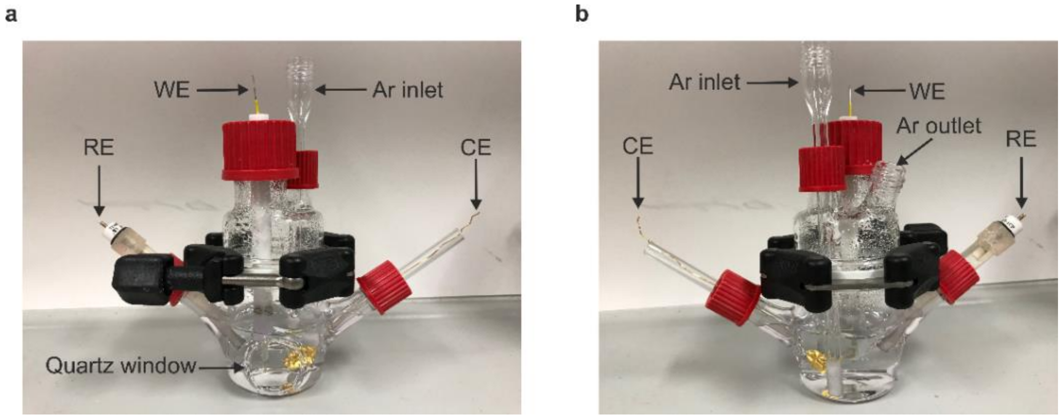

Figure S13. Photographs of (a) the front and (b) back side of home-made reactor for three-electrode measurements. The light impinges from the front side of the quartz window.

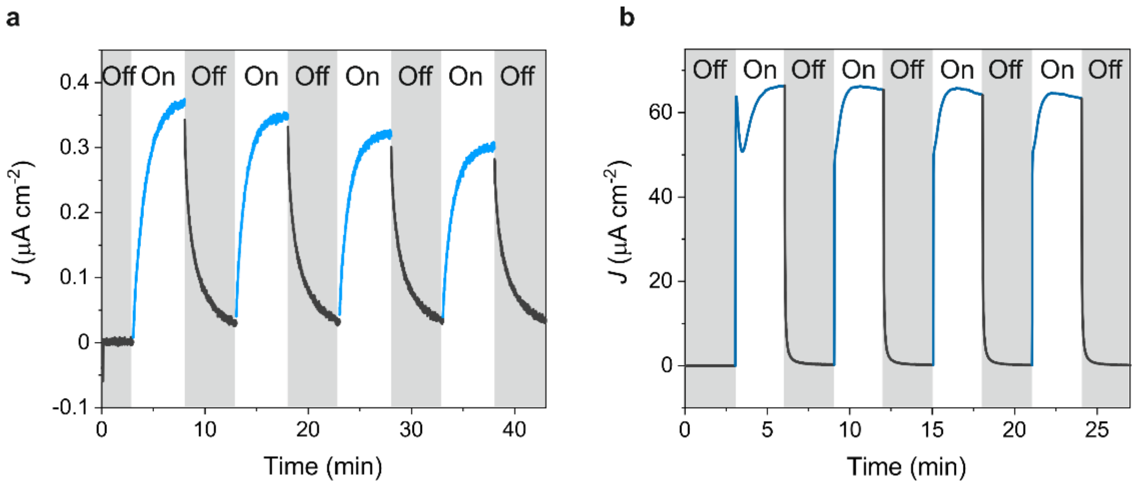

Figure S14. Photocurrent of $NbWO_6$ photoanodes under (a) 1 sun and (b) 365 nm UV illumination in oxygen-free 1M LiCl in the presence of MeOH. A pronounced decrease of the maximum photocurrent over time is observed under 1 sun illumination, while very little decrease is seen under 365 nm UV illumination (see also Fig. S33). Chronoamperometry (CA) experiments were performed under an applied potential of -0.1 V.

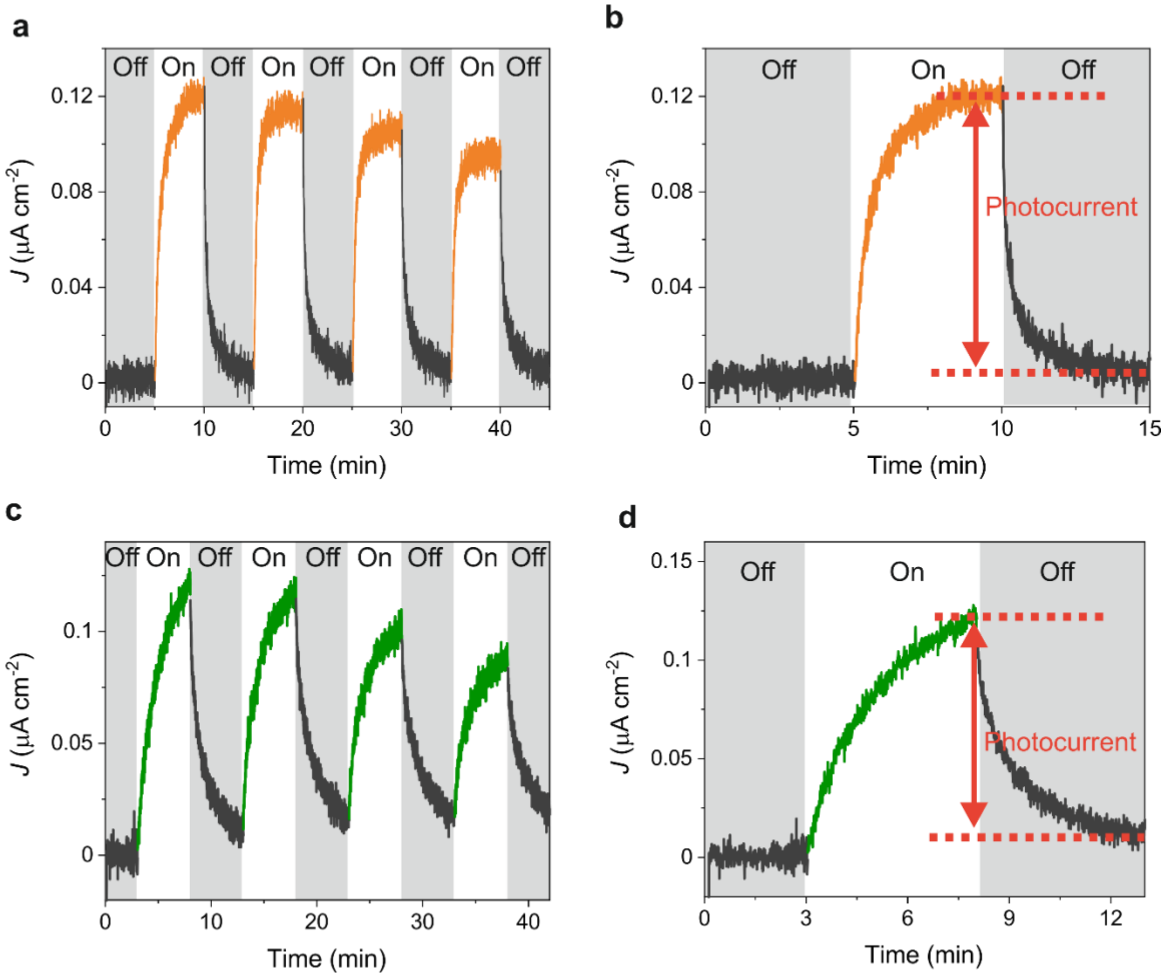

Figure S15. Photocurrent of $NbWO_6$ photoanodes in oxygen-free (a) 1M LiCl in the presence of 10 mM 4-MBA and (c) 1M LiCl electrolyte in pure H_2O under 1 sun illumination. The first off/on/off cycles of (a, c) are shown in (b, d), respectively. CA experiments were performed under an applied potential of -0.1 V.

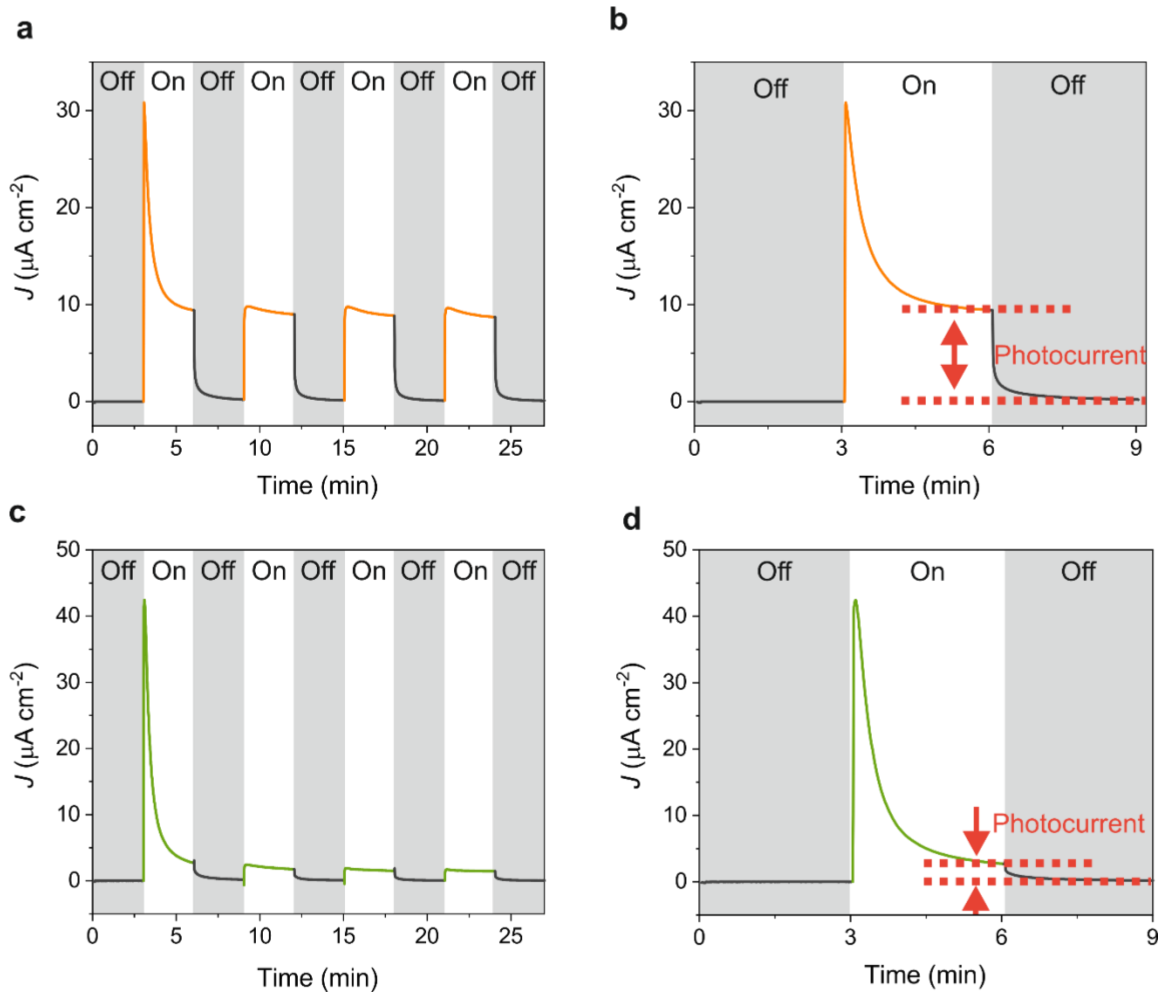

Figure S16. Photocurrents of $NbWO_6$ electrodes in oxygen-free (a) 1M LiCl in the presence of 10 mM 4-MBA, and (c) 1M LiCl electrolyte in H_2O under 365 nm UV illumination. The first off/on/off cycles of (a, c) are shown in (b, d), respectively. CA experiments were performed with an applied potential of -0.1 V.

The photocurrent transient spikes may be caused by the discrepancy between the fast carrier generation, recombination, and slow surface reaction dynamics.[11] They confirm the lower efficiency of 4-MBA and H_2O donors compared to MeOH.

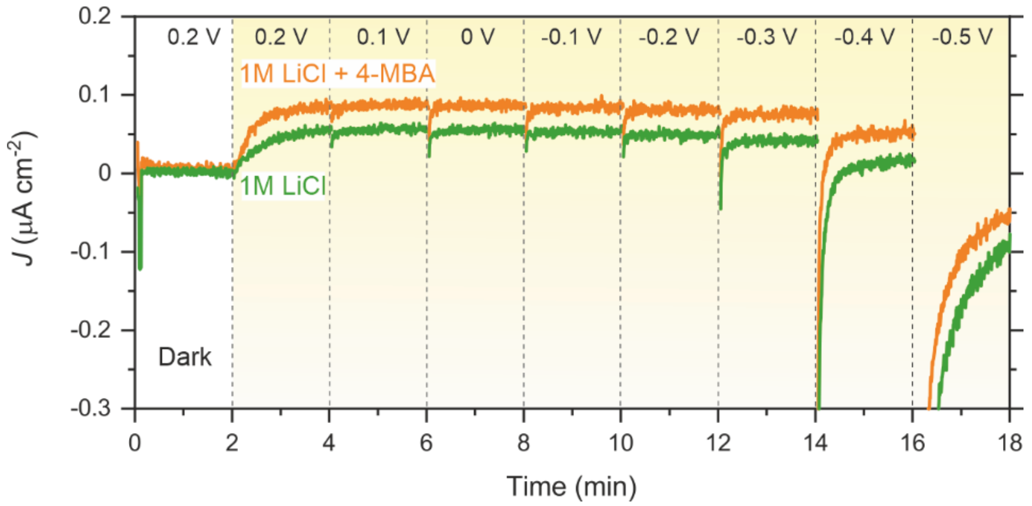

Figure S17. Photocurrents at different potentials in different donors under 1 sun illumination. A negligible current is observed in the dark under an applied potential of 0.2 V for both cases. Like in Fig. 4e, a positive current flow is observed between 0.2 V to -0.3 V upon illumination, indicating the photo-generated electrons in the CB were extracted. Further increasing the potential to negative applied potentials decreases the current due to the decrease in the driving force for electron extraction.

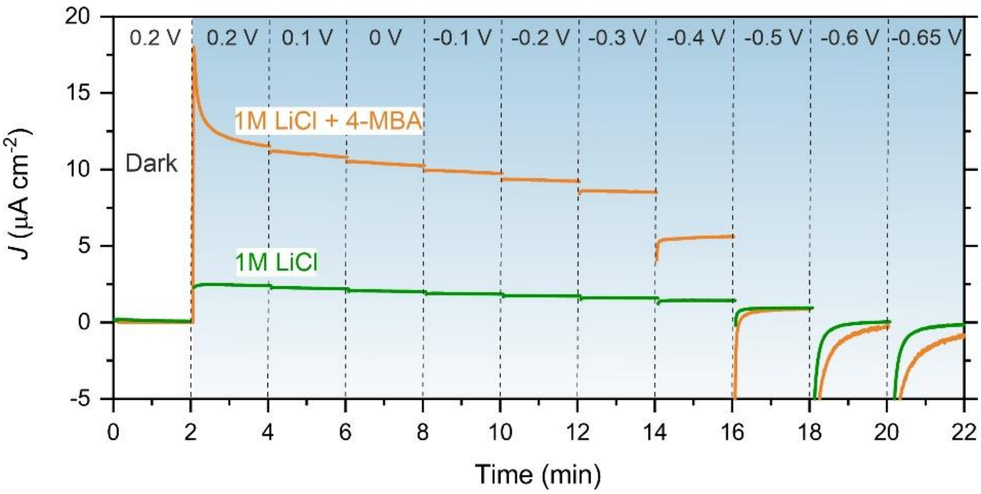

Figure S18. Photocurrents at different potentials in different donors under 365 nm UV illumination. A negligible current is observed in the dark under an applied potential of 0.2 V for both cases. Upon illumination, a positive current flow is observed between 0.2 V to -0.4 V, indicating the photo-generated electrons in the CB were extracted. A further decrease in potential decreases the current due to the decrease in the driven force. The positive photocurrent generated in 1M LiCl electrolyte suggest that water can be used as donor, but less efficiently than 4-MBA and MeOH.

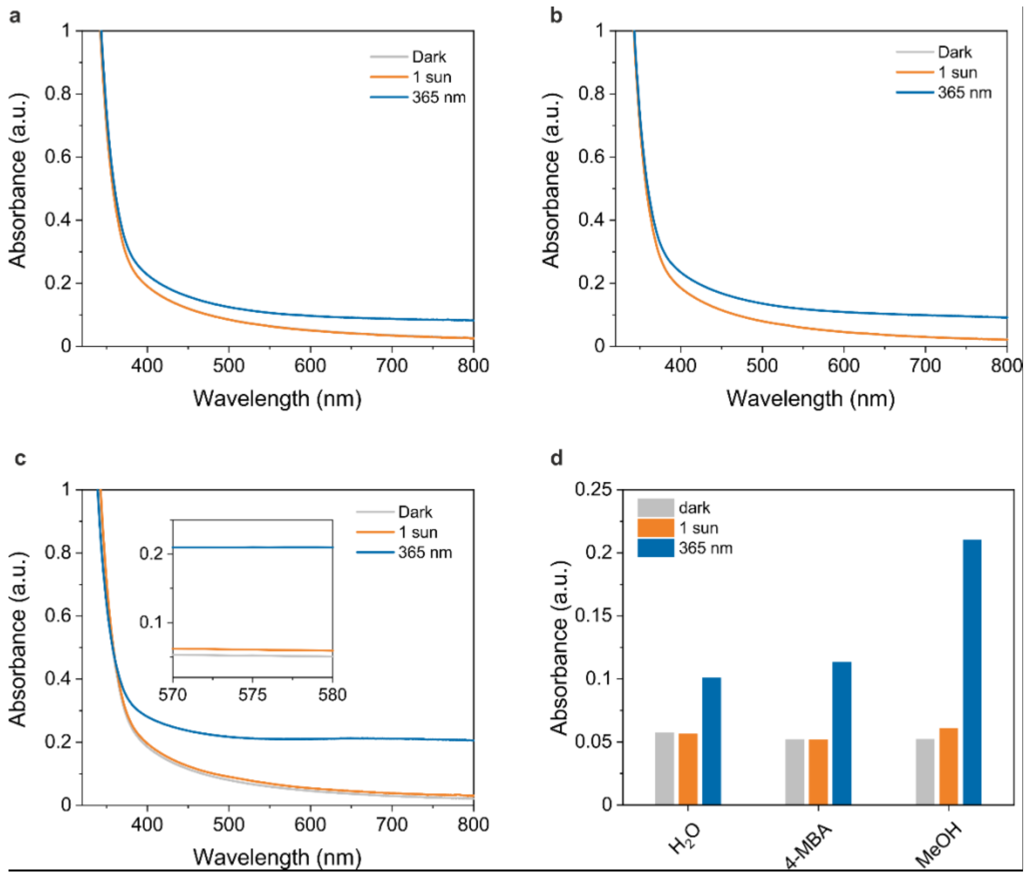

Figure S19. UV-Vis absorbance spectra of oxygen-free $NbWO_6$ nanosheet suspension in the presence of (a) H_2O, (b) 4-MBA, and (c) MeOH donors under light illumination for 10 min. The insert in (c) shows the magnified region between 570 nm to 580 nm. (d) Summary of the dependence of absorption intensity at a wavelength of 575 nm as a function of the different donors.

We measured UV-Vis absorption spectra to quantify the blue color change after light illumination. The absorbance at a wavelength of 575 nm in the presence of MeOH donor under 365 nm UV illumination is higher than for 4-MBA in water, while the absorption intensity in water is the lowest. These results further confirm that MeOH shows higher efficiency for hole quenching than 4-MBA and water.

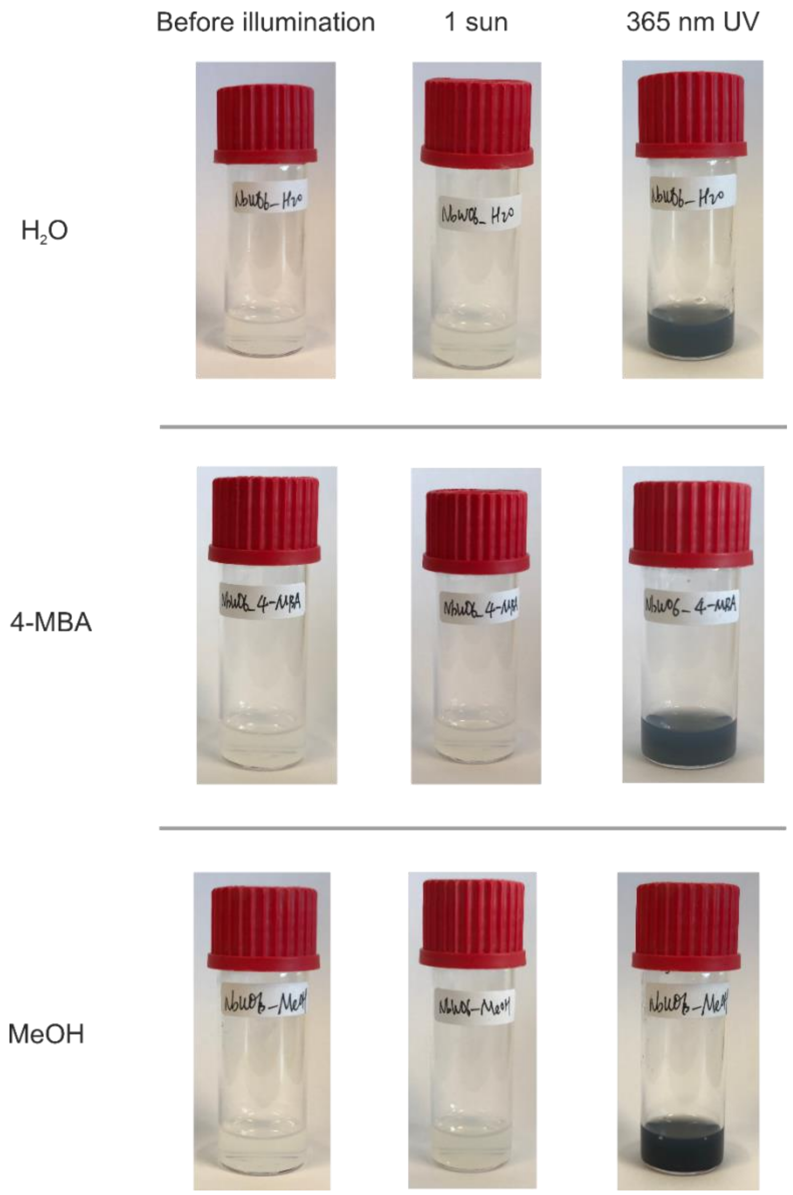

Figure S20. Color change of oxygen-free $NbWO_6$ nanosheet suspensions in the presence of different donors before and after light illumination for 10 min.

The suspension shows light milky color before illumination. After 1 sun illumination, no obvious color change was observed. However, the suspension exhibits significant color change under 365 nm UV illumination. The suspension with MeOH as donor shows dark blue color, while the one in H_2O and 4-MBA donors also show blue color but less intense than for MeOH. The color change indicates that $NbWO_6$ is able to store photogenerated electrons in the conduction band under 365 nm UV illumination, while the storage ability under 1 sun illumination is significantly lower.

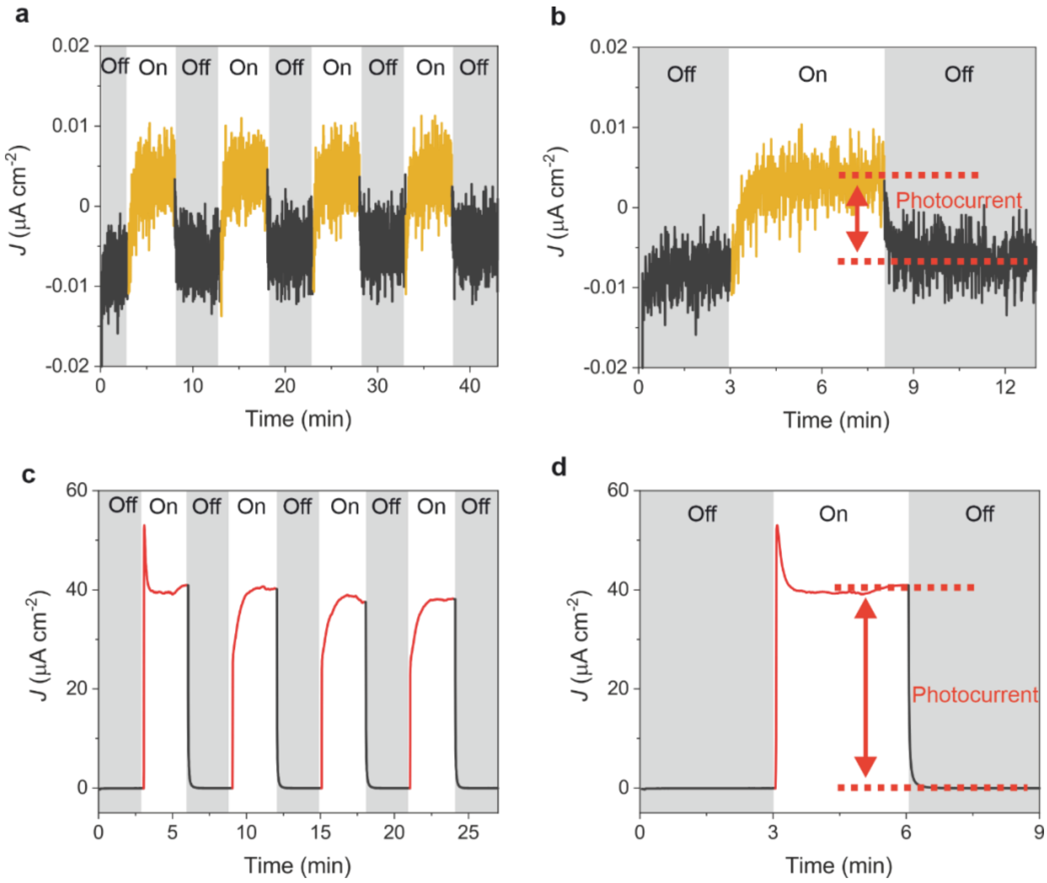

Figure S21. Photocurrents of $NbWO_6$ electrodes in oxygen-rich 1M LiCl in the presence of MeOH (10 vol%) donor under (a) 1 sun and (c) 365 nm UV illumination. The first off/on/off cycles of (a, c) are shown in (b, d), respectively. CA experiments were performed with an applied potential of -0.1 V.

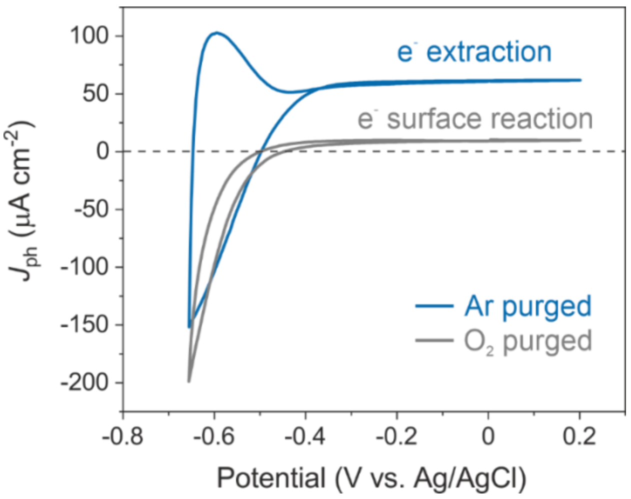

Figure S22. CV sweep in 1M LiCl and MeOH electrolyte under 365 nm UV illumination and continuous Ar and oxygen purging, respectively. The scan rate is 10 mV s^{-1}.

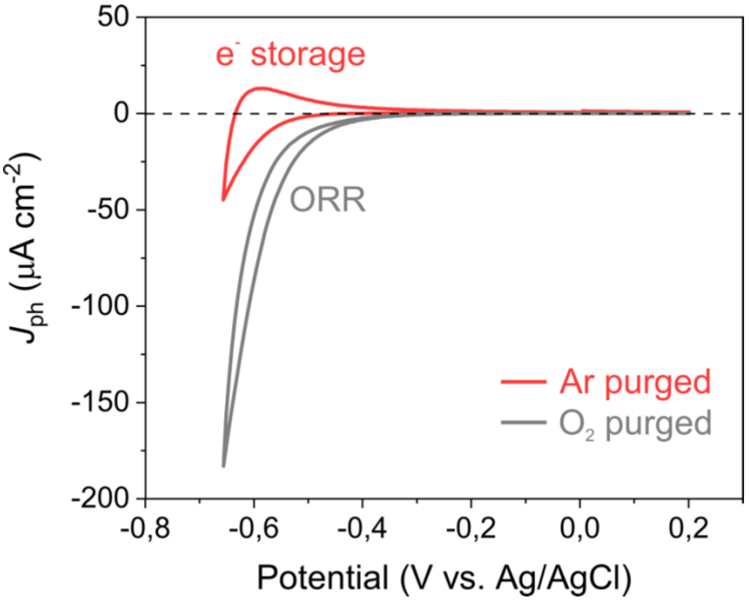

Figure S23. Dark CV sweep measurement shows reversible electron storage and release in continuous Ar purging, and the oxygen reduction reaction (ORR) in oxygen rich environment. The results highlight the importance of oxygen-free electrolyte.

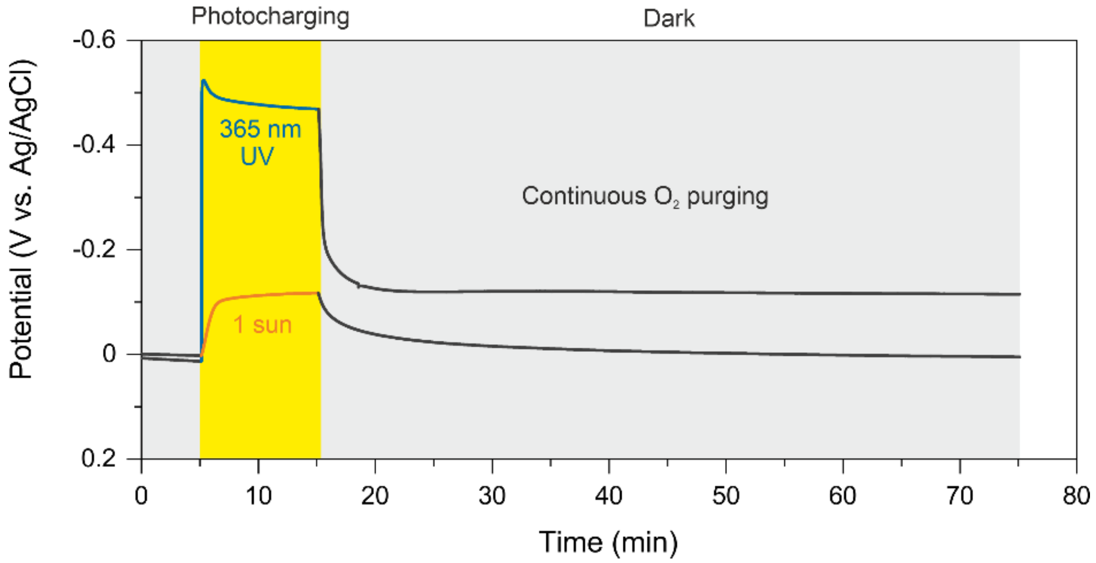

Figure S24. OCP of $NbWO_6$ electrodes in oxygen-rich 1 M LiCl in the presence of MeOH donor under 1 sun and 365nm UV illumination. The electrode was illuminated for 10 min and left in the dark for 1 h. The presence of oxygen would scavenge photo-generated electrons from $NbWO_6$, which decreases the OCP of the electrodes during light charge. The OCP drops dramatically when light charging is stopped, indicating the electron scavenging side reaction by oxygen, which quickly consumes the photo-generated electrons.

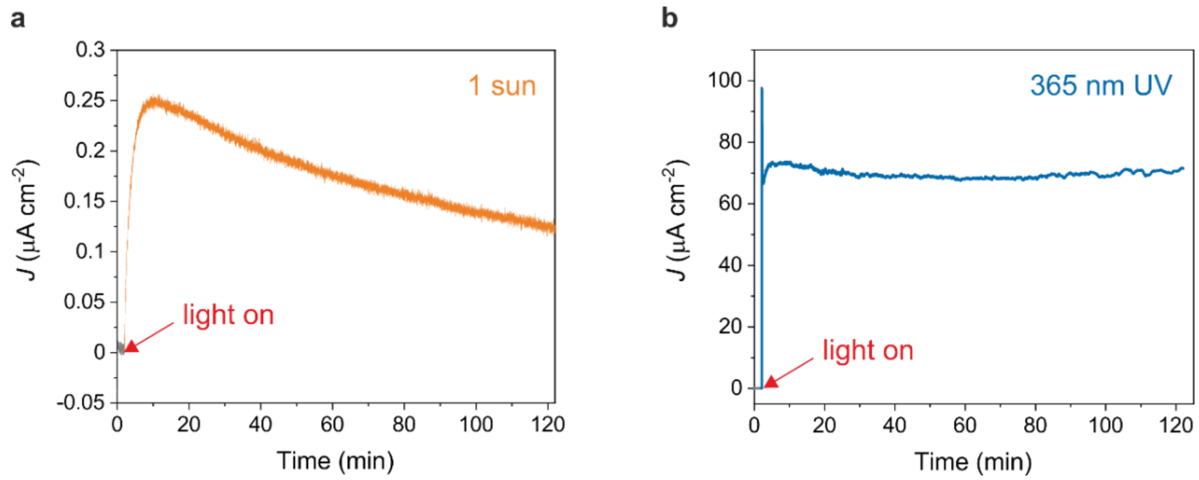

Figure S25. Chronoamperometry photocurrent stability measurements under (a) 1 sun and (b) 365 nm UV illumination in oxygen-free 1M LiCl and MeOH electrolyte. The applied potential is -0.1 V. A decrease in photocurrent is observed under 1 sun illumination, while almost no decrease is observed under 365 nm UV illumination, indicating more stable charge storage under UV illumination.

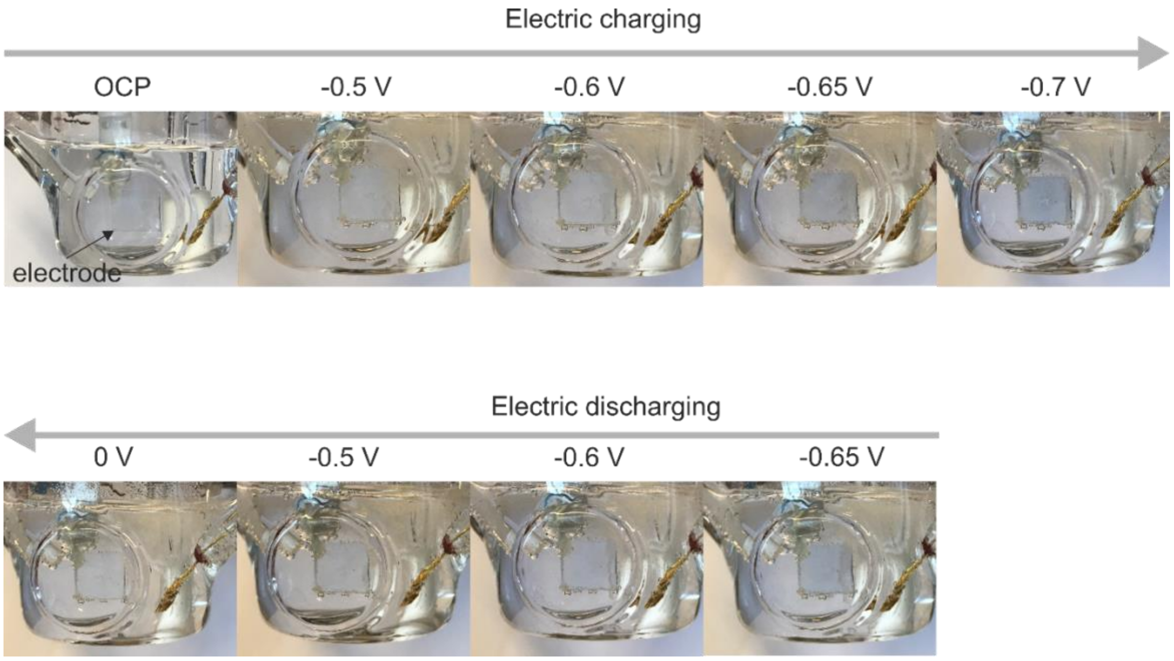

Figure S26. Color change of the $NbWO_6$ electrode during electric charge and discharge in oxygen-free 1M LiCl electrolyte.

The electrode shows high transparence at OCP conditions. There is no color change when charging to -0.5 V. When further charging the electrode to -0.65 V, the electrode exhibits light blue color. A significant color change is observed when charging the electrode to -0.7 V. The electrode shows similar light blue color when discharging it to -0.65 V. The electrode becomes highly transparent when discharging to -0.5 V, indicating a highly reversible charge and discharge processes.

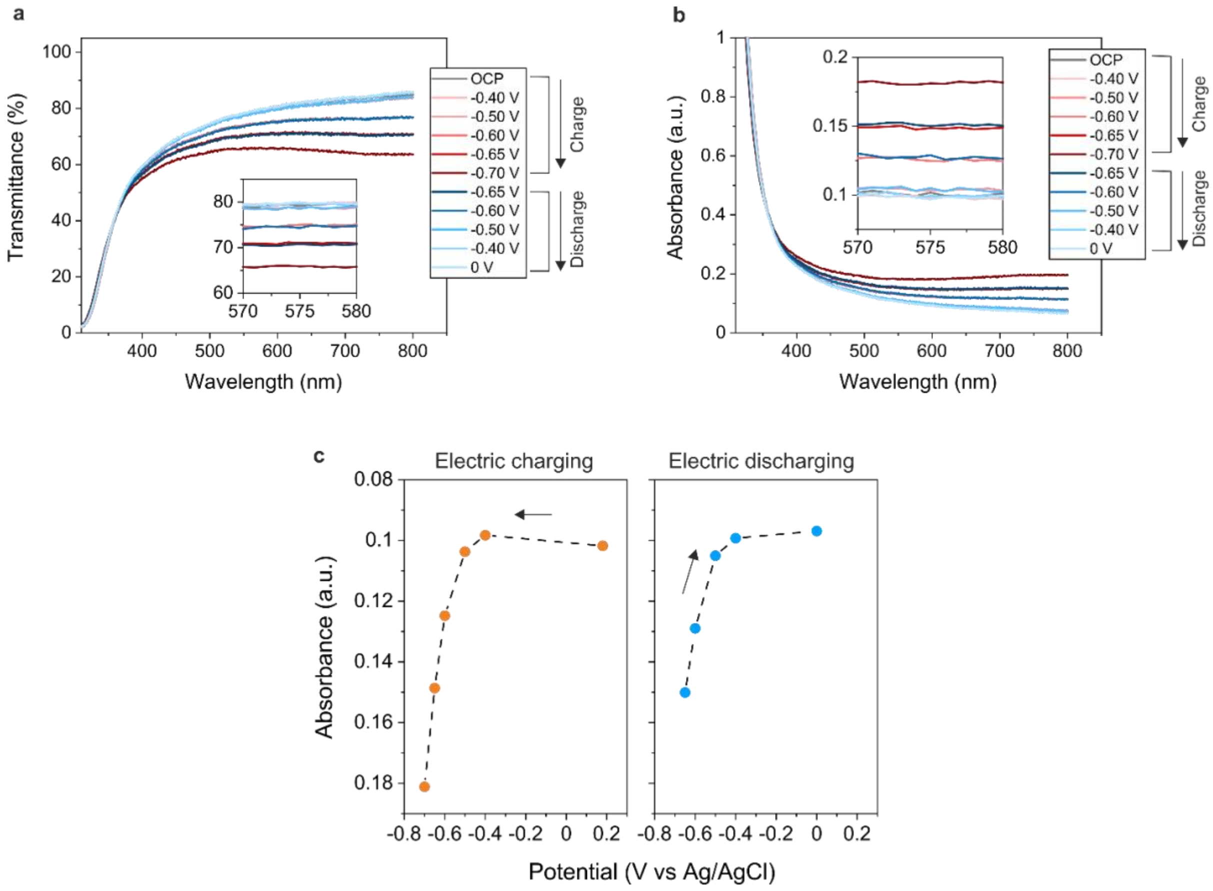

Figure S27. (a) Operando UV-vis transmittance spectra and corresponding (b) absorbance spectra of a $NbWO_6$ photoanode under different applied bias in oxygen-free 1M LiCl. The inserts in (a) and (b) show the magnified region between 570 nm and 580 nm. (c) The absorbance intensity at 575 nm under different applied potentials. The left plot shows the intensity trend during electric charging from OCP (0.18 V) to -0.7 V. The right plot shows the intensity trend during electric discharging from -0.65 V to 0 V. The arrows indicate the potential scanning direction.

As shown in Fig. S26, the $NbWO_6$ electrode in oxygen-free 1M LiCl changes to blue color under the electric charge and discharge processes. To quantify the relationship between the intensity of the blue color and applied potential, we performed in-situ UV-vis transmittance spectra. As shown in Fig. S27a-b, the transmittance starts to decrease and the absorption starts to increase when the electrode is charged to -0.6 V from OCP, indicating charge storage. The transmittance continuously decreases when the electrode is charged to more negative potential, suggesting charge accumulation in the electrode. Upon discharge, the transmittance starts to increase when the electrode is discharged to 0 V. A summary of

absorption intensity at 575 nm upon electric charge and discharge is shown in Fig. S27c. It is noteworthy that the electrode absorbance intensity exhibits high reversibility.

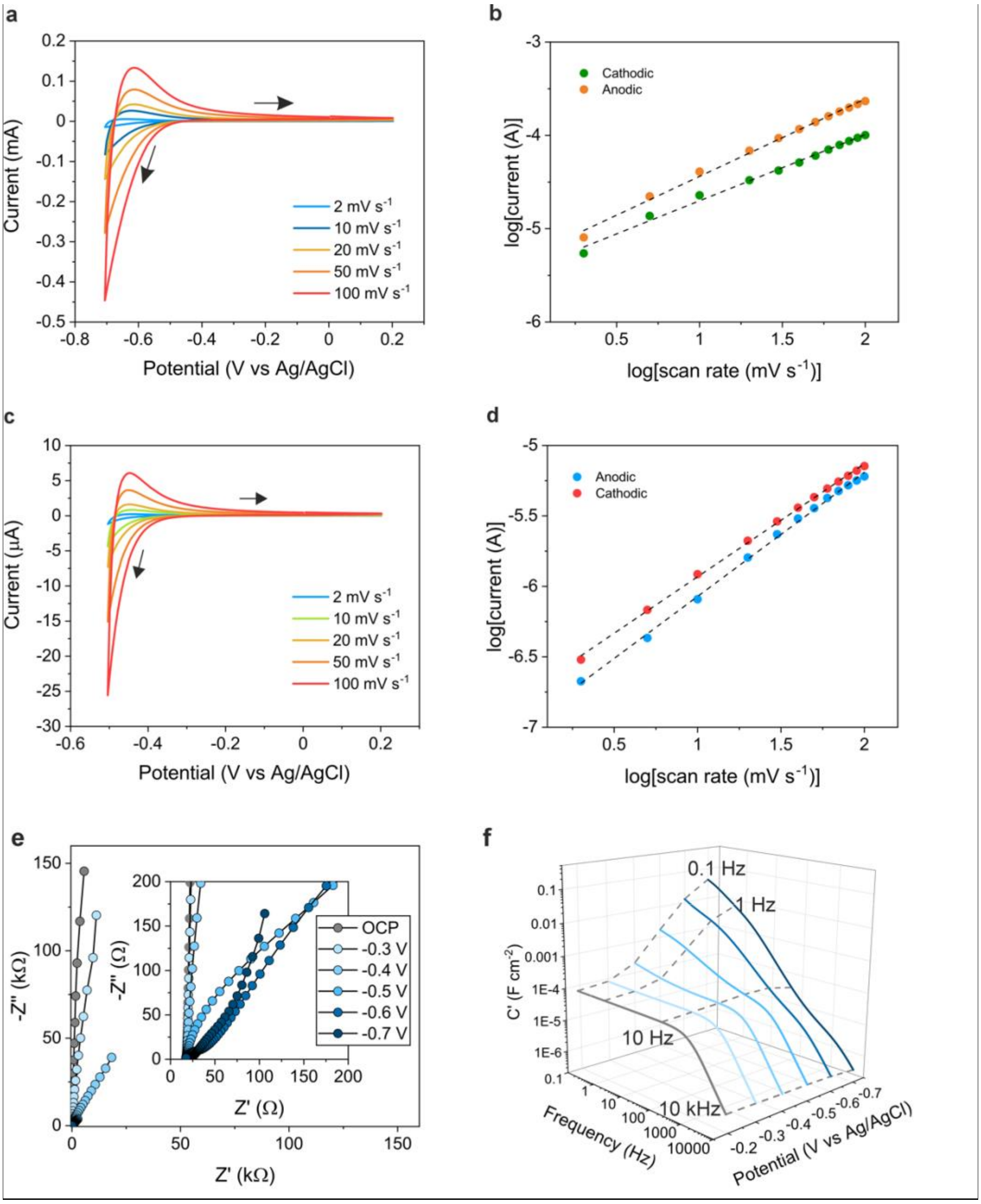

Figure S28. **Kinetic study of the electrochemical behavior of a $NbWO_6$ electrode**. CV scanning from 2 to 100 mV s^{-1} in the potential window between (a) 0.2 to -0.7 V and (**c**) 0.2 to -0.5 V. The log(*i*) vs log(*v*) plots for anodic and cathodic currents at (b) -0.65 V and (d) -0.45 V. *b*-values are 0.83 and 0.71 for cathodic and anodic currents in (b), and 0.80 and 0.88 for cathodic and anodic currents in (d), respectively. (e) Nyquist plots under different applied bias potentials. The insert shows the magnified high-frequency region. (f) 3D Bode plot of the capacitance vs frequency and potential in (e). The dashed lines connect the C' vs potential at a specific frequency. The electrolyte used in (a, c, e) is oxygen-free 1 M LiCl.

We study the electrochemical properties of the $NbWO_6$ thin film electrode fabricated by means of drop casting on FTO by carrying out a kinetic cyclic voltammetry (CV) study. A broad anodic peak around -650 mV vs Ag/AgCl (saturated KCl) is found in the CV when scanning from 2 to 100 mV s^{-1} in oxygen-free 1M LiCl (Fig. S28a), which likely originates from a Faradaic redox reaction of $NbWO_6$. The electrode color which changes to blue under electric charging further confirms a redox reaction of $NbWO_6$ to take place (Fig. S26-27). The color change is likely due to the partial change in oxidation state of tungsten from 6+ to 5+ during the charge/discharge process. The CV shape suggests that partially desolvated Li^+ ions intercalate into the $NbWO_6$ electrode.[12] Fig. S28b shows the plot of both anodic and cathodic currents at -650 mV against the scan rate. Assuming the currents obey a power law yields[13]:

$$i = av^b$$

where *a* and *b* are the adjustable values. The *b* value of 0.5 indicates that the current is diffusion controlled, while a value of 1 indicates a surface controlled process, which is capacitive. The currents show a linear relationship with the scan rate, with values of the characteristic parameter *b* of 0.83 and 0.71 for cathodic and anodic currents (Fig. S28b), respectively. Similarly, the *b* values are 0.80 and 0.88 for cathodic and anodic currents in Fig. S28d, respectively. The experimentally found *b* values indicate that the energy storage mechanism is a combination of surface capacitive and diffusion-like processes, i.e. pseudocapacitive behavior, which is typical for 2D materials.[14]

To gain insights into the nature of charge transfer and mechanism of charge storage in the $NbWO_6$ thin film electrode, electrochemical impedance spectroscopy (EIS) under different applied bias potentials were performed in the dark (Fig. S28e). All Nyquist plots show similar series resistance R_C of approximately 16 Ω. However, there is a difference in the charge transfer mechanism under different applied bias potentials. The near-vertical imaginary parts for OCP, -0.3 V and -0.4 V in the low frequency region indicate a capacitive-like charge storage mechanism, while the Nyquist plots for -0.5 V, -0.6 V and -0.7 V exhibit a clear 45° Warburg-type impedance in the mid-frequency range, indicating a diffusion-limited process. A Bode-type plot is further used to understand the energy storage mechanism of $NbWO_6$ (Fig. S28f). The real capacitance C’ exhibits almost constant values in the low frequency range from OCP to -0.4 V, which is characteristic of a capacitive process. In contrast, C’ increases significantly with increasing negative bias potential, indicating a diffusion-limited process.[14]

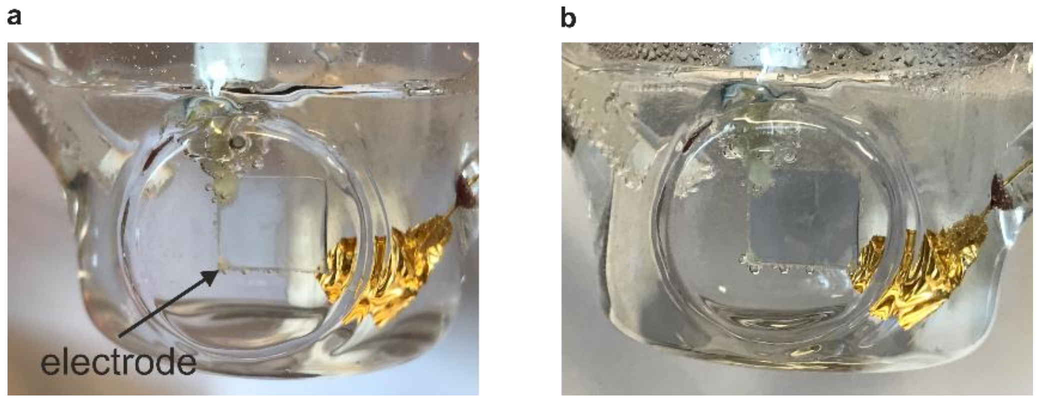

Figure S29. Color change of a $NbWO_6$ photoanode under (a) 1 sun and (b) 365 nm UV illumination for 10 min in oxygen-free 1M LiCl in the presence of MeOH (10 vol%). There is no obvious color change under 1 sun illumination, while the one under 365 nm UV illumination exhibits blue color.

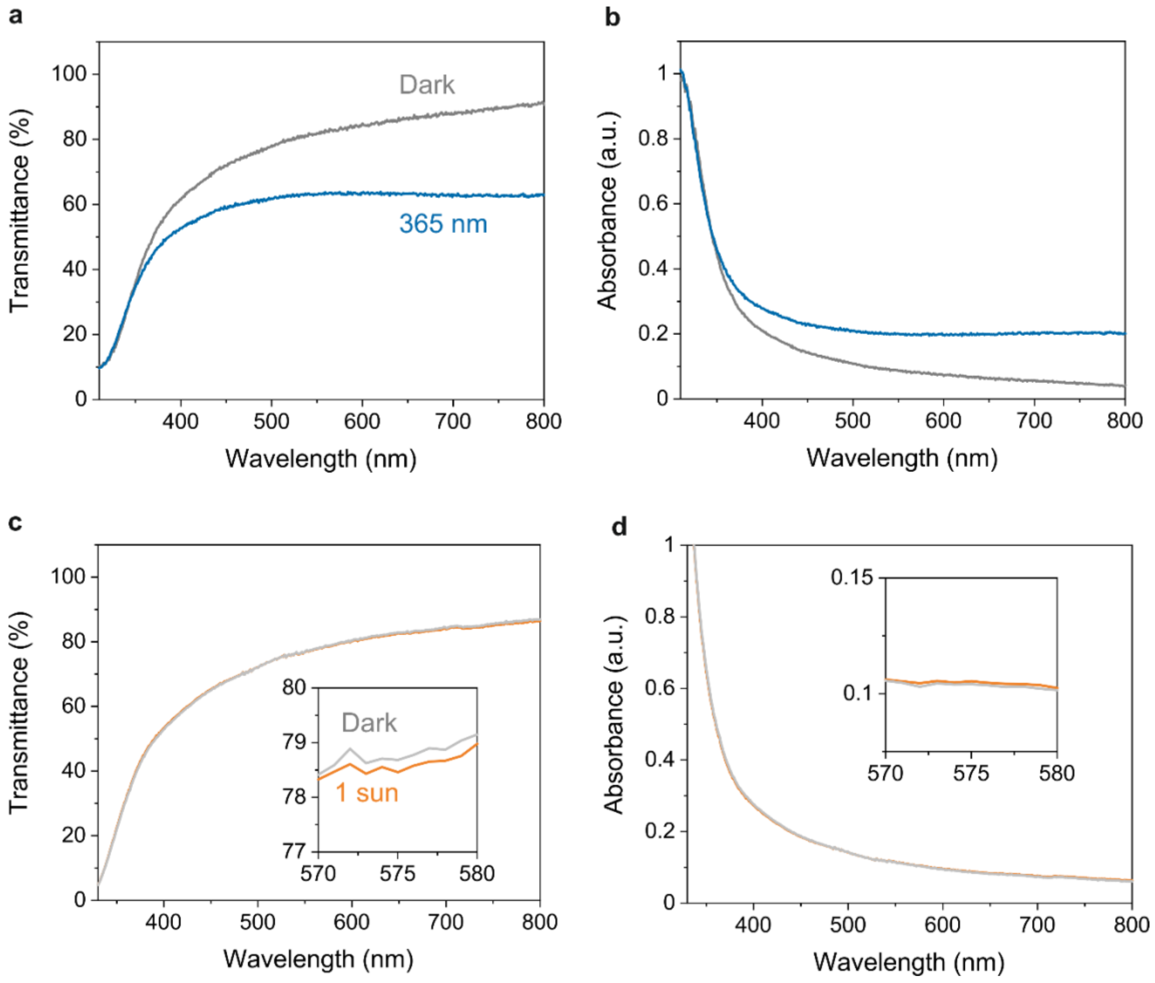

Figure S30. UV-vis transmittance spectra and its corresponding absorbance spectra for a $NbWO_6$ photoanode under (a, b) 365 nm UV illumination and (c, d) 1 sun illumination for 10 min. The electrolyte is oxygen-free 1M LiCl in the presence of 10 vol% MeOH. The inserts in (c) and (d) show the magnified region between 570 nm and 580 nm.

As shown in Fig. S29, the photoanode shows a change towards blue color upon 365 nm UV illumination. The UV-vis transmittance/absorbance spectra (Fig. S30a-b) further confirm the change in blue color, while there is no obvious absorption intensity change at 575 nm under 1 sun illumination (Fig. S30d).

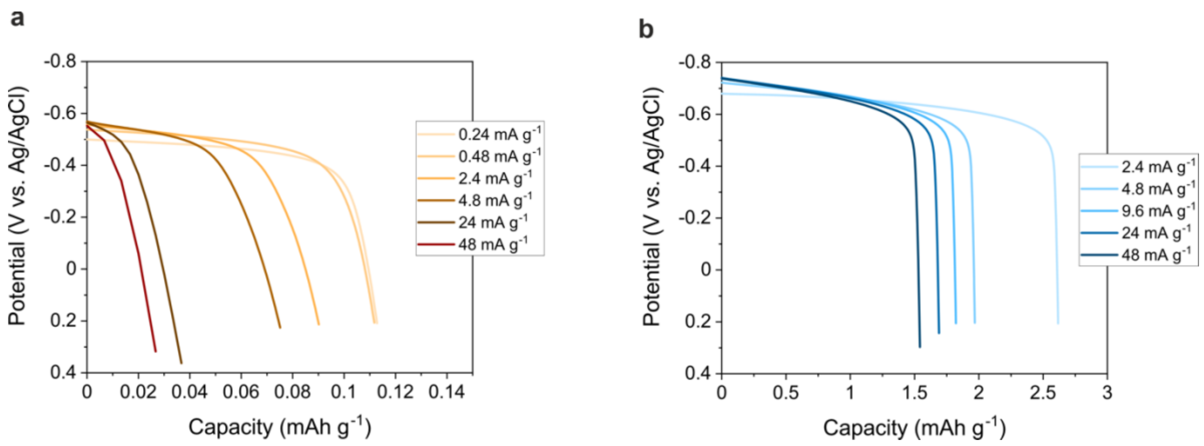

Figure S31. Electrical discharge profiles at different discharge current densities after illumination under (a) 1 sun and (b) 365 nm UV light for 10 min in 1M LiCl and MeOH electrolyte under continuous Ar purging.

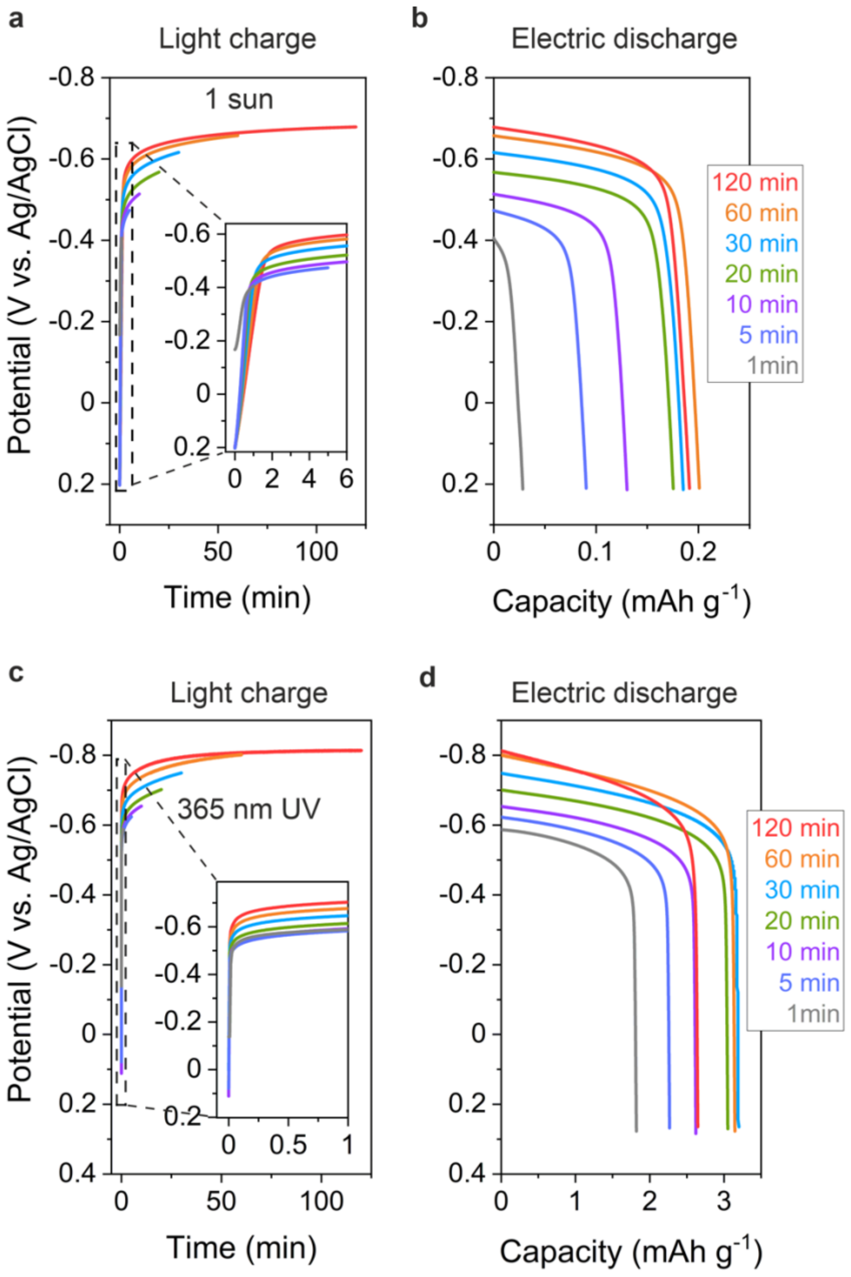

Figure S32. The light charging profiles under different illumination times for (a) 1 sun and (c) 365 nm UV illumination and the corresponding electric discharge profiles. The discharge current densities are (b) 0.48 mA g^{-1} and (d) 4.8 mA g^{-1}. Inserts show the OCP under illumination at the beginning, i.e. up to one minute. The electrolyte is oxygen-free 1M LiCl with the presence of MeOH.

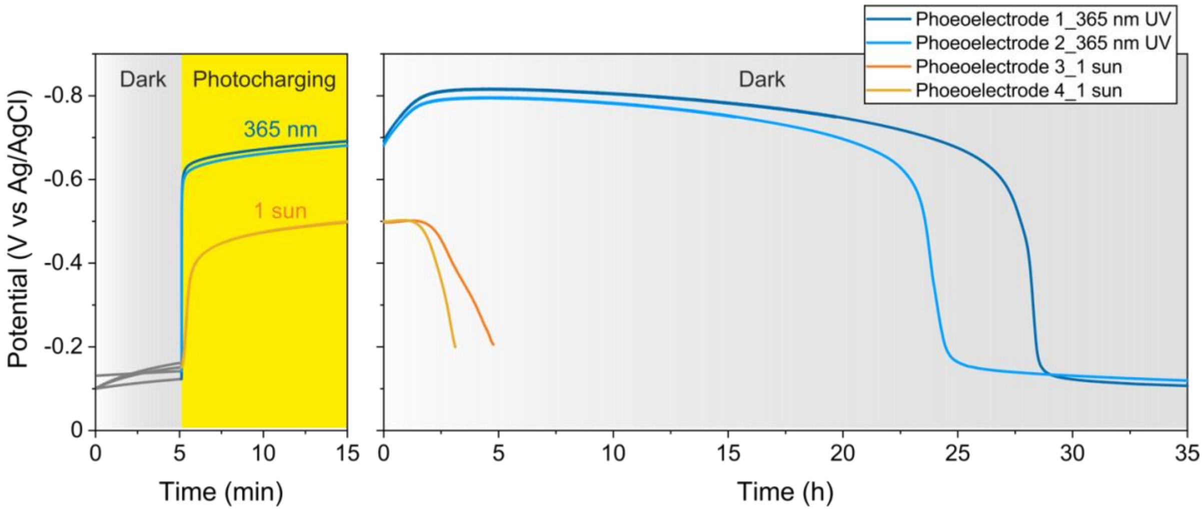

Figure S33. Reproducibility measurements of OCP stability of different batches of $NbWO_6$ photoanodes. The photoanodes were illuminated under light for 10 min in 1M LiCl and MeOH electrolyte under continuous Ar purging. Then the OCP of photoanodes were left in the dark. The yellow lines in the left image indicate 1 sun illumination, the blue lines indicate 365 nm UV illumination.

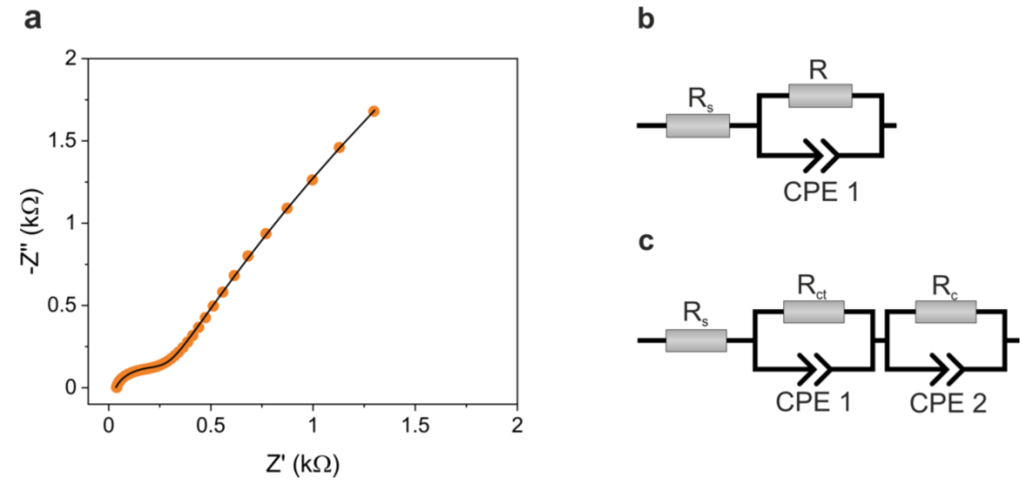

Figure S34. (a) Nyquist plot of $NbWO_6$ photoanode under 1 sun illumination. The fitted equivalent circuit models used for the $NbWO_6$ photoanode (b) in the dark and (c) under 1 sun and 365 nm UV illumination in Fig. 5d.

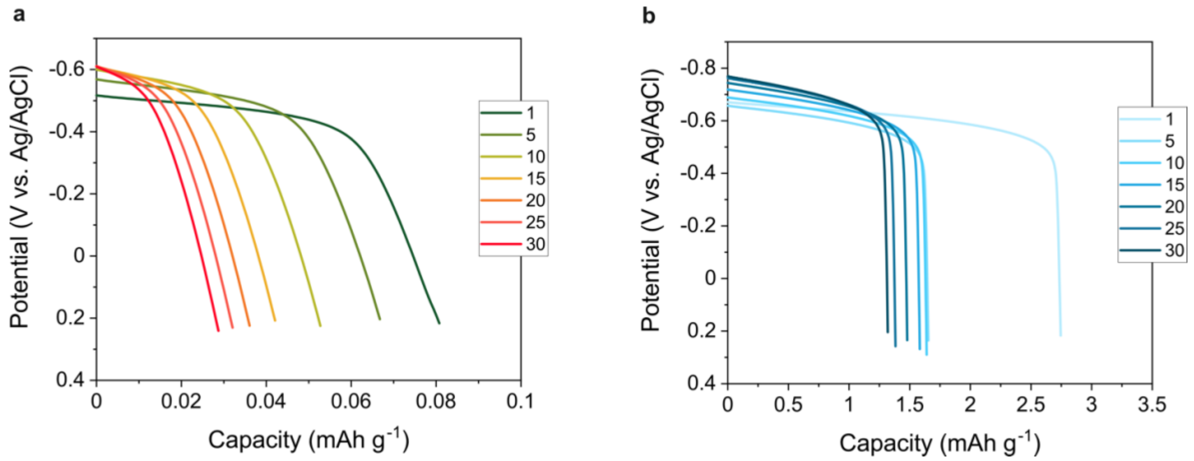

Figure S35. Electric discharge profiles after (a) 1 sun illumination for 10 min and (b) 365 nm UV illumination for 5 min for different cycles. The discharge current densities are (a) 4.8 mA g^{-1} and (b) 24 mA g^{-1}.

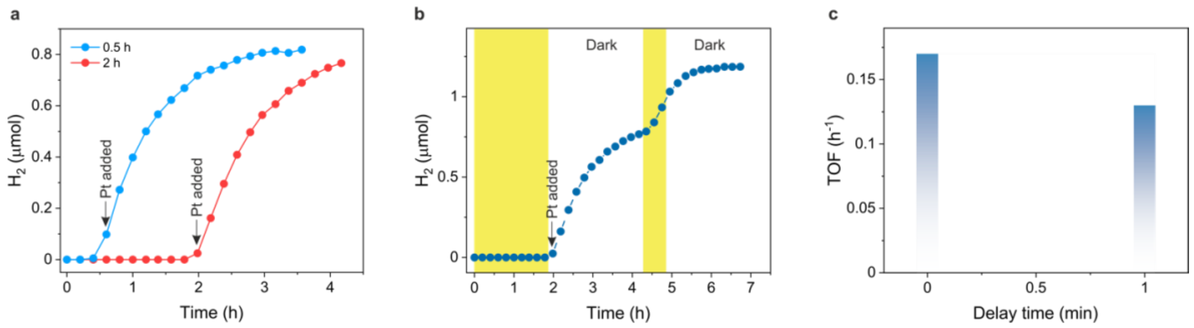

Figure S36. (a) The hydrogen amount as a function of illumination time. (b) Long-term measurement for dark hydrogen generation. The yellow regions correspond the light illumination. (c) Histogram plot shows the turnover frequency (TOF) after 365 UV illumination for 30 min without delay time and with 1 h delay time.

Table S3. Fitted results of the EIS measurement in the dark as shown in Fig. 5d.

Parameters	Value	Error (%)
R_S (Ω)	27.16	4.1
R_C (Ω)	7.72E+6	1.3
CPE Q 1	1.02E-5	0.0098
CPE Alpha 1	0.99	0.01

Table S4. Fitted results of the EIS measurement under 1 sun and 365 nm UV illumination as shown in Fig. 5d.

	1 sun		365 nm UV	
Parameters	Value	Error (%)	Value	Error (%)
R_S (Ω)	35.24	7.5	16.17	4.4
R_{CT} (Ω)	222.80	2.5	290.95	7.4
CPE Q 1	7.26E-05	9.8	1.73E-3	3.1
CPE Alpha 1	0.79	2.6	0.53	1.8
R_C (Ω)	1.7505E+4	3.5	3258.37	10
CPE Q 2	6.64E-04	0.14	4.16E-3	3
CPE Alpha 2	0.712	0.43	0.92	2

References

1 Fourquet, J. L., Le Bail, A. & Gillet, P. A. $LiNbWO_6$: Crystal structure of its two allotropic forms. *Mater. Res. Bull.* **23**, 1163-1170 (1988).

2 Biesinger, M. C. Accessing the robustness of adventitious carbon for charge referencing (correction) purposes in XPS analysis: Insights from a multi-user facility data review. *Appl. Surf. Sci.* **597**, 153681 (2022).

3 Fairley, N. *et al.* Systematic and collaborative approach to problem solving using X-ray photoelectron spectroscopy. *Appl. Surf. Sci. Adv.* **5**, 100112 (2021).

4 Taberna, P. L., Simon, P. & Fauvarque, J. F. Electrochemical characteristics and impedance spectroscopy studies of carbon-carbon supercapacitors. *J. Electrochem. Soc.* **150**, A292-A300 (2003).

5 Lau, V. W. *et al.* Dark Photocatalysis: Storage of Solar Energy in Carbon Nitride for Time-Delayed Hydrogen Generation. *Angew. Chem. Int. Ed. Engl.* **56**, 510-514 (2017).

6 Kröger, J. *et al.* Interfacial Engineering for Improved Photocatalysis in a Charge Storing 2D Carbon Nitride: Melamine Functionalized Poly(heptazine imide). *Adv. Energy Mater.* **11**, 2003016 (2020).

7 Coelho, A. A. TOPAS and TOPAS-Academic: an optimization program integrating computer algebra and crystallographic objects written in C++. *J. Appl. Crystallogr.* **51**, 210-218 (2018).

8 Cheary, R. W. & Coelho, A. A fundamental parameters approach to X-ray line-profile fitting. *J. Appl. Crystallogr.* **25**, 109-121 (1992).

9 Rietveld, H. M. A profile refinement method for nuclear and magnetic structures. *J. Appl. Crystallogr.* **2**, 65-71 (1969).

10 Le Bail, A., Duroy, H. & Fourquet, J. L. Ab-initio structure determination of $LiSbWO_6$ by X-ray powder diffraction. *Mater. Res. Bull.* **23**, 447-452 (1988).

11 Lian, Z. *et al.* Efficient Self-Driving Photoelectrocatalytic Reactor for Synergistic Water Purification and H_2 Evolution. *ACS Appl. Mater. Interfaces* **12**, 44731-44742 (2020).

12 Fleischmann, S. *et al.* Continuous transition from double-layer to Faradaic charge storage in confined electrolytes. *Nat. Energy* **7**, 222-228 (2022).

13 Lindström, H. *et al.* Li+ Ion Insertion in TiO_2 (Anatase). 2. Voltammetry on Nanoporous Films. *J. Phys. Chem. B* **101**, 7717-7722 (1997).

14 Fleischmann, S. *et al.* Pseudocapacitance: From Fundamental Understanding to High Power Energy Storage Materials. *Chem. Rev.* **120**, 6738-6782 (2020).
